# Supplementary material for: ChAdOx1 nCoV-19 vaccine elicits monoclonal antibodies with cross-neutralizing activity against SARS-CoV-2 viral variants
Source: Cell Rep. 2022 Apr 15;39(5):110757. doi: 10.1016/j.celrep.2022.110757 (PMC9010245; doi:10.1016/j.celrep.2022.110757)
Supplement: Document S1. Figures S1–S5 and Table S1 [file mmc1.pdf]

Cell Reports, Volume *39*

Supplemental information

ChAdOx1 nCoV-19 vaccine elicits monoclonal antibodies with cross-neutralizing activity against SARS-CoV-2 viral variants

Jeffrey Seow, Carl Graham, Sadie R. Hallett, Thomas Lechmere, Thomas J.A. Maguire, Isabella Huettner, Daniel Cox, Hataf Khan, Suzanne Pickering, Rebekah Roberts, Anele Waters, Christopher C. Ward, Christine Mant, Michael J. Pitcher, Jo Spencer, Julie Fox, Michael H. Malim, and Katie J. Doores

ChAdOx1 nCoV-19 (AZD1222) vaccine elicits monoclonal antibodies with potent cross-neutralizing activity against SARS-CoV-2 viral variants

Jeffrey Seow,[1]* Carl Graham,[1]* Sadie R. Hallett,[1] Thomas Lechmere,[1] Thomas J.A. Maguire,[1] Isabella Huettner,[1] Daniel Cox,[1] Hataf Khan,[1] Suzanne Pickering,[1] Rebekah Roberts,[2] Anele Waters,[2] Christopher C. Ward,[1] Christine Mant,[3] Michael J. Pitcher,[4] Jo Spencer,[4] Julie Fox,[1,2] Michael H. Malim,[1] Katie J. Doores[1]#

[1] Department of Infectious Diseases, School of Immunology & Microbial Sciences, King's College London, London, UK.
[2] Harrison Wing, Guys and St Thomas' NHS Trust, London, UK.
[3] Infectious Diseases Biobank, Department of Infectious Diseases, School of Immunology and Microbial Sciences, King's College London, London, UK.
[4] Peter Gorer Department of Immunobiology, School of Immunology & Microbial Sciences, King's College London, London, UK.

* These authors contributed equally
# Corresponding author: katie.doores@kcl.ac.uk

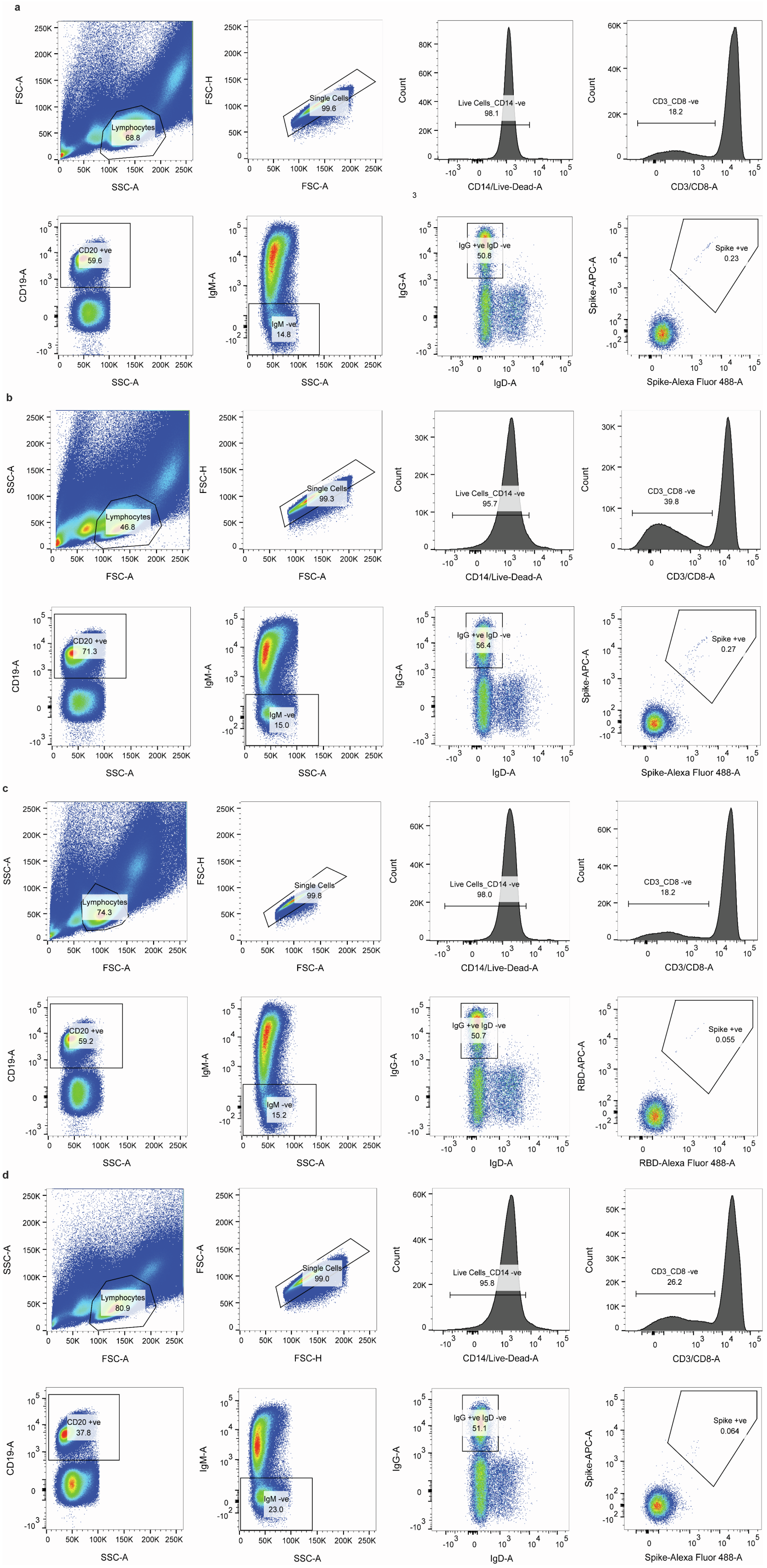

Supplementary Figure 1: FACS sorting strategy to isolate Spike and RBD reactive mAbs following vaccination with AZD1222, related to **Figure 1**. Sorting of Spike reactive IgG+ B cells from VA14 at **A)** 4-months (TP1) and **B)** 9-months (TP2) post vaccine booster. **C)** Sorting of RBD reactive IgG+ B cells from VA14 at 4-months post vaccine booster. **D)** Staining of PBMC collecting pre-COVID-19 from a healthy control.

a

Name	Protein + Epitope	VH-GENE	JH-GENE	DH-GENE	VL-GENE	JL-GENE	CDRH3 Length	CDRL3 Length	Neutralzation	% Identity Heavy	% Identity Light
VA014_09	RBD	IGHV1-18	IGHJ3	IGHD3-10	IGKV3-11	IGKJ4	16	8	Yes	95.41	96.55
VA014_50	RBD	IGHV1-18	IGHJ3	IGHD3-10	IGKV3-11	IGKJ4	16	8	Yes		
VA014_R39	RBD	IGHV4-34	IGHJ6	IGHD4-17	IGKV1-5	IGKJ2	18	8	Yes	87.1	94.36
VA014_26	RBD	IGHV4-34	IGHJ6	IGHD4-17	IGKV1-5	IGKJ2	18	8	Yes		
VA014_27	RBD	IGHV5-51	IGHJ3	IGHD2-2	IGKV1-39	IGKJ2	23	9	Yes	95.38	95.03
VA014_67	RBD	IGHV5-51	IGHJ6*	IGHD2-2	IGKV1-39	IGKJ2	23	9	Yes		

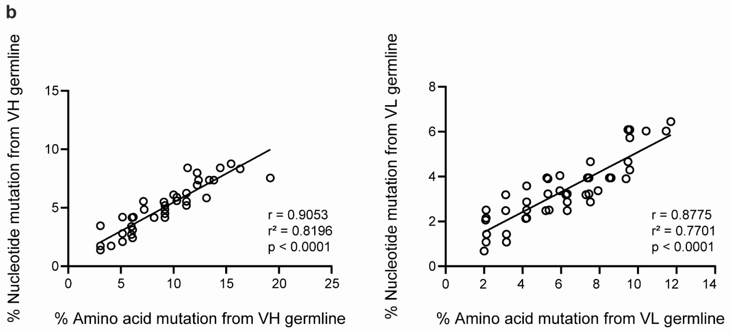

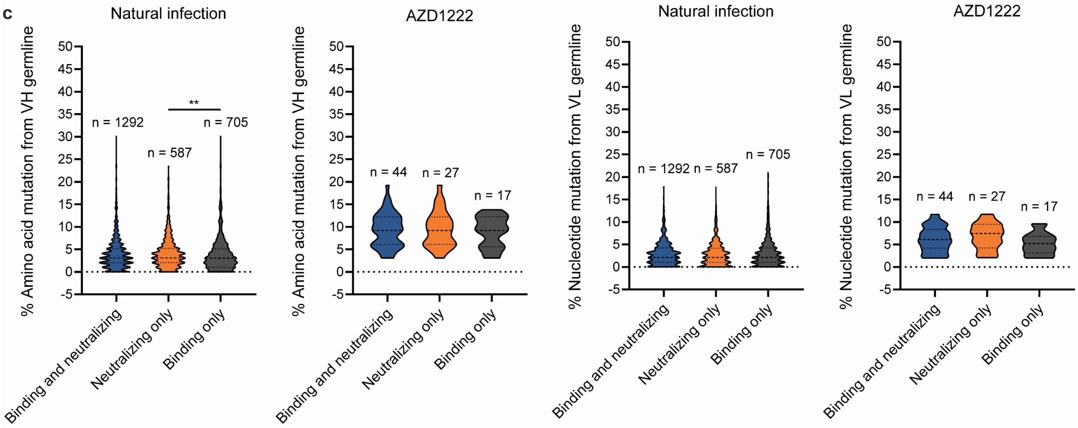

Supplementary Figure 2: Sequence analysis of AZD1222 elicited mAbs, related to **Figure 3**. **A)** Clonally related mAbs isolated from VA14. **B)** Correlation between the % nucleotide mutation and % amino acid mutation for VH and VL germline for AZD1222 elicited mAbs (Spearman correlation, two-tailed, r). A linear regression was used to calculate the goodness of fit (r^2). **C)** Divergence from germline (based on amino acid alignments) for VH and VL genes for Spike reactive mAbs arising from natural infection and AZD1222 vaccination. Spike reactive mAbs have been separated based on their binding and/or neutralizing properties. D'Agostino & Pearson tests were performed on each dataset to determine normality. Based on the result, either a Kruskal-Wallis test with Dunn's multiple comparison post hoc test or an ordinary one-way ANOVA with Turkey's multiple comparison post hoc test was performed. *p<0.0332, **p<0.0021, ***p<0.0002 and ****<0.0001.

A: RBD mAbs with RBD ELISA

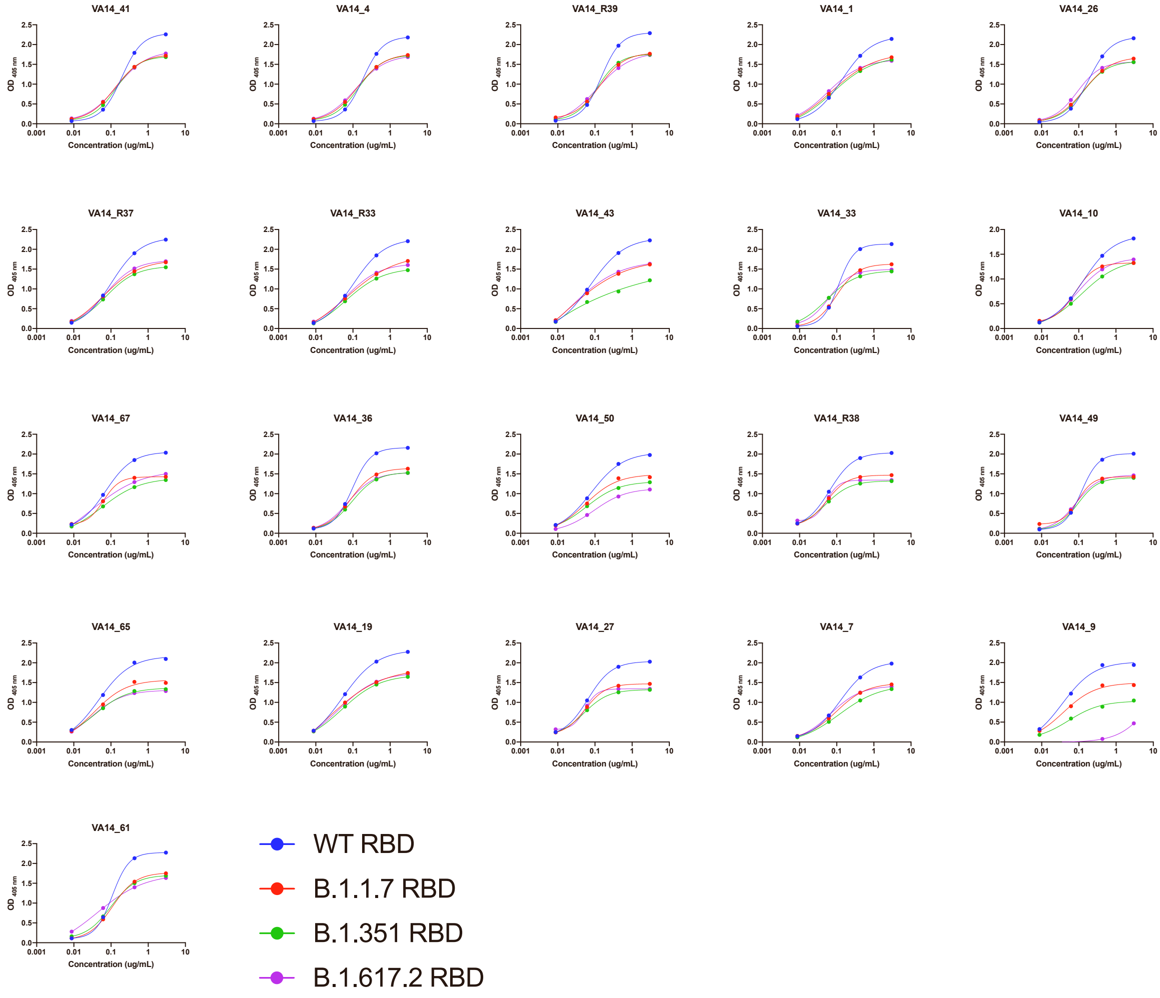

B: NTD mAbs with S1 ELISA

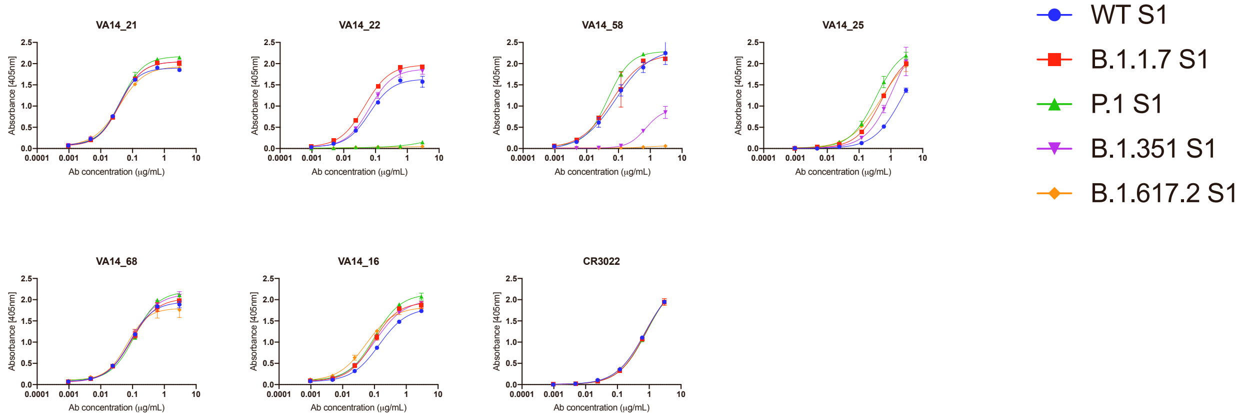

Supplementary Figure 3: Binding of AZD1222 neutralizing antibodies to RBD or S1 from SARS-CoV-2 variants of concern, related to **Figure 4** and **Figure 5**. **A)** Binding of RBD-specific nAbs to recombinant RBD from WT, alpha, beta and delta by ELISA. **B)** Binding of NTD-specific nAbs to recombinant S1 from WT, alpha, beta, gamma and delta by ELISA.

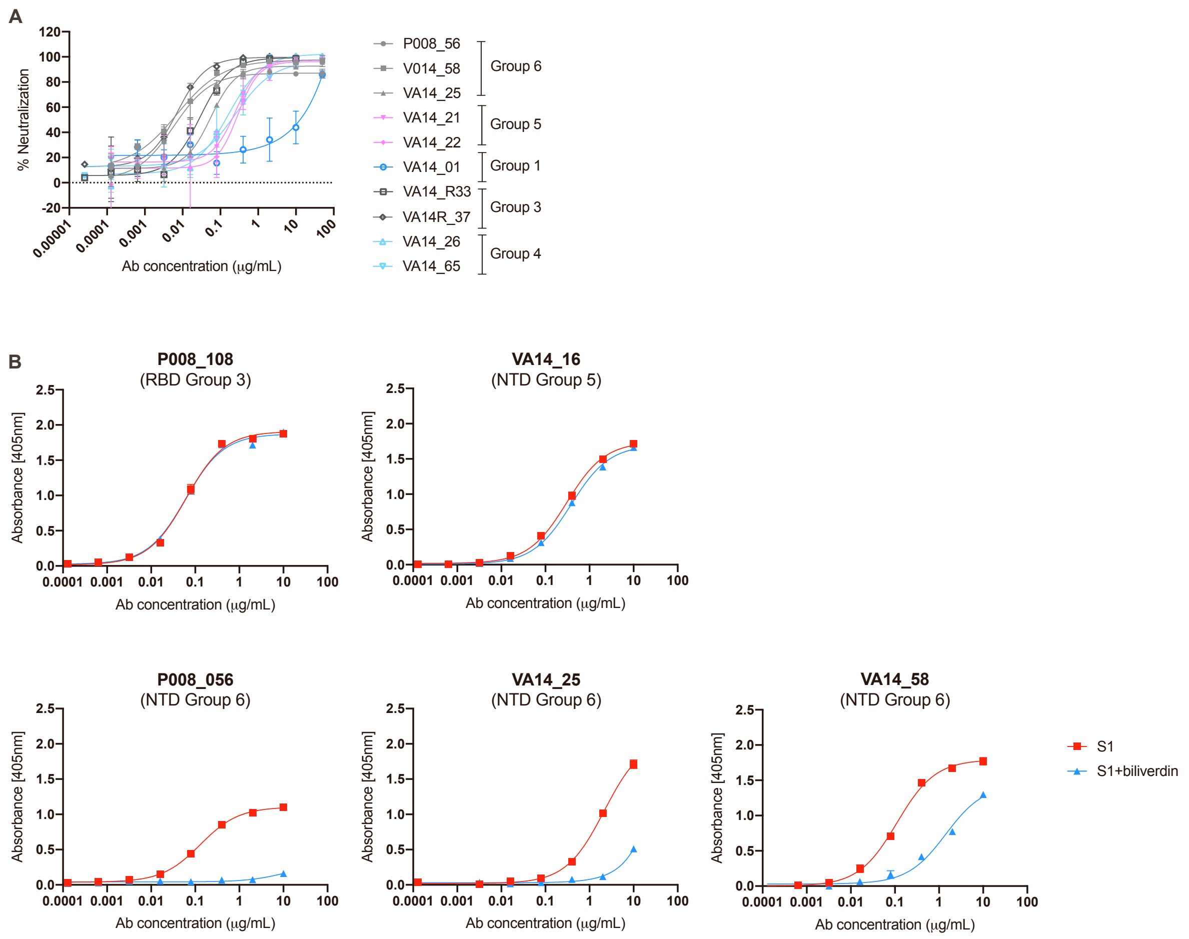

Supplemental Figure 4: Characterisation of NTD-specific Group 6 nAbs, related to **Figure 5**. **A)** Neutralization of SARS-CoV-2 (England 02/2020/407073) infectious virus using Vero-E6 TMPRSS2 cells. Experiments were performed in duplicate. nAbs are colour coded by group. **B)** Binding of VA14 NTD Group 6 mAbs to S1 in the presence (blue) and absence (red) of Biliverdin. RBD Group mAb P008_108 and NTD Group 5 mAb VA14_16 are used as negative controls. NTD Group 6 mAb P008_056 is used as a positive control (Rosa et al., 2021).

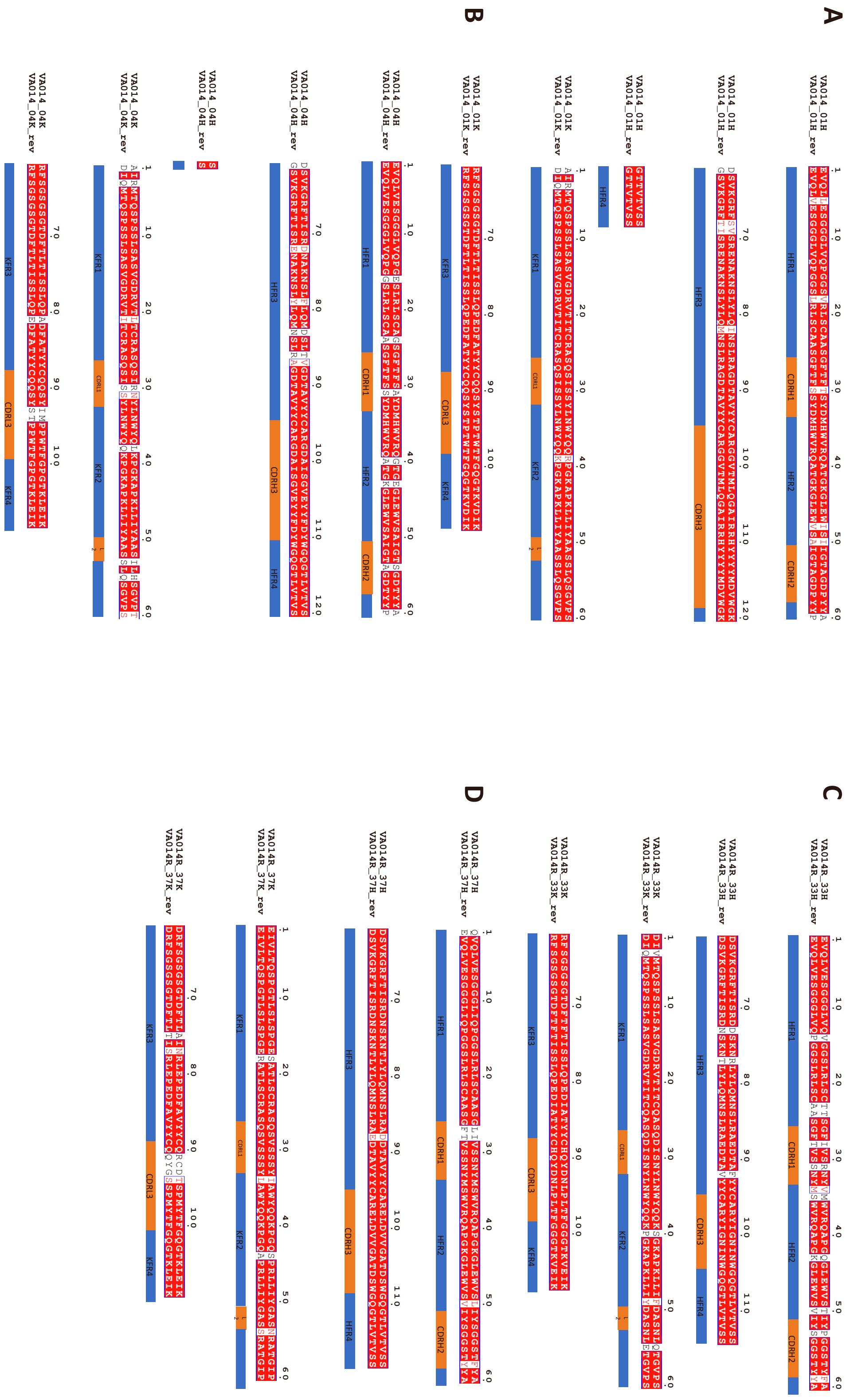

Supplementary Figure 5: Amino acid alignments for germline reverted heavy and light chains, related to **Figure 6**. **A)** VA14_01, **B)** VA14_04, **C)** VA14R_33, **D)** VA14R_37. Top germline V-gene hits were determined using the International Immunogenetics Information System (IMGT) database (Lefranc et al., 1999). The sequence alignment was determined by Clustal Omega (Sievers et al., 2011) and plotted with ESPRIPT (Robert and Gouet, 2014). CDR1, 2 and 3 regions are highlighted. Related to **Figure 6**.

	WT IC_{50}	B.1.1.7 IC_{50}	P.1 IC_{50}	B.1.351 IC_{50}	B.1.617.2 IC_{50}	B.1.1.529 IC_{50}	ACE2 Competition	Specificity	Competition Group	VH	VL
VA014_04	0.42	0.78	0.56	0.65	0.82	5.63	96	RBD	Group 1	IGHV3-13	IGKV1-39
VA014_41	0.84	1.46	0.69	1.26	1.52	48.12	91	RBD	Group 1	IGHV3-30	IGKV1-39
VA014_01	7.34	5.18	2.94	8.17	11.08	44.68	66	RBD	Group 1	IGHV3-13	IGKV1-39
VA014R_39	0.070	0.091	0.079	0.046	0.055	>50	99	RBD	Group 1	IGHV4-34	IGKV1-5
VA014_26	0.21	0.17	0.093	0.11	0.11	>50	98	RBD	Group 2	IGHV4-34	IGKV1-5
VA014R_37	0.024	0.0094	0.0038	0.0013	0.018	0.0020	99	RBD	Group 3	IGHV3-53	IGKV3-20
VA014R_33	0.010	0.020	0.0040	0.0011	0.018	0.0050	99	RBD	Group 3	IGHV3-66	IGKV1-33
VA014_43	0.029	0.0044	0.0037	0.0097	0.017	0.164	99	RBD	Group 3	IGHV3-53	IGKV1-9
VA014_33	0.056	0.031	0.0056	0.0056	0.013	0.010	90	RBD	Group 4	IGHV3-74	IGLV6-57
VA014_36	0.025	0.020	0.013	0.0062	0.020	1.26	72	RBD	Group 4	IGHV4-59	IGKV1-33
VA014_65	0.047	0.038	0.047	0.27	>50	0.19	59	RBD	Group 4	IGHV3-21	IGKV1-39
VA014_19	0.79	0.32	0.24	1.15	0.29	0.65	55	RBD	Group 4	IGHV3-15	IGKV1-39
VA014_49	3.06	0.91	1.31	3.04	2.16	5.98	55	RBD	Group 4	IGHV3-30	IGKV1-33
VA014R_38	0.038	0.040	0.053	0.092	0.040	0.12	45	RBD	Group 4	IGHV3-43	IGKV3-20
VA014_50	0.031	0.024	0.66	1.39	>50	1.01	45	RBD	Group 4	IGHV1-18	IGKV3-11
VA014_10	0.78	0.13	3.70	4.95	0.25	5.08	45	RBD	Group 4	IGHV3-23	IGKV1-33
VA014_67	0.029	0.022	0.049	0.29	0.055	0.52	43	RBD	Group 4	IGHV5-51	IGKV1-39
VA014_07	0.28	0.19	0.46	1.16	0.86	3.08	43	RBD	Group 4	IGHV4-39	IGKV3-20
VA014_27	0.24	0.18	0.16	0.73	1.98	0.96	34	RBD	Group 4	IGHV5-51	IGKV1-39
VA014_09	0.027	0.019	11.9	>50	>50	13.08	28	RBD	Group 4	IGHV1-18	IGKV3-11
VA014_61	0.071	0.016	0.0069	0.0060	0.032	0.0020	96	RBD	Group 4	IGHV1-69	IGKV4-1
VA014_21	1.22	0.84	13.4	5.17	14.07	3.17	58	NTD	Group 5	IGHV1-69	IGKV4-1
VA014_22	0.13	0.53	>50	0.78	>50	0.95	51	NTD	Group 5	IGHV3-48	IGKV1D-12
VA014_58	>50	0.0085	0.026	>50	>50	>50	28	NTD	Group 6	IGHV1-8	IGKV3-11
VA014_25	>50	0.052	0.093	>50	>50	>50	19	NTD	Group 6	IGHV1-69	IGKV3-15
VA014_47	0.86	32.37	>50	1.38	1.08	0.79	59	S only	Group 7	IGHV3-30	IGKV3-20
VA014_16	0.14	0.051	0.14	0.22	0.027	1.59	23	NTD	Group 8	IGHV3-48	IGKV1-17
VA014_68	0.39	0.075	10.19	0.48	0.067	1.86	19	NTD	Group 8	IGHV4-59	IGKV4-1

Supplementary Table S1: Binding and neutralization properties of isolated mAbs, related to **Figure 2** and **Figure 5**.
