# Supplementary material for: ChAdOx1 nCoV-19 vaccine elicits monoclonal antibodies with cross-neutralizing activity against SARS-CoV-2 viral variants
Source: Cell Rep. 2022 Apr 15;39(5):110757. doi: 10.1016/j.celrep.2022.110757 (PMC9010245; doi:10.1016/j.celrep.2022.110757)
Supplement: Document S2. Article plus supplemental information [file mmc2.pdf]

Cell Reports

Article

ChAdOx1 nCoV-19 vaccine elicits monoclonal antibodies with cross-neutralizing activity against SARS-CoV-2 viral variants

Graphical abstract

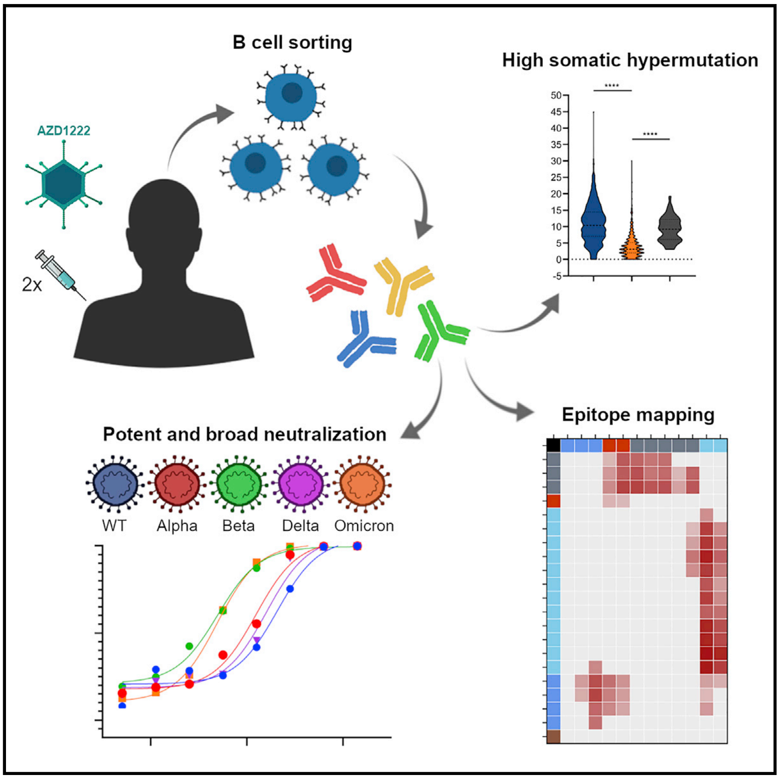

Highlights

- AZD1222 vaccination elicits monoclonal antibodies with potent neutralization
- Vaccine antibodies target epitopes overlapping infection-induced antibodies
- Vaccine antibodies have broad neutralization activity against VOCs, including Omicron
- Despite low serum neutralization, Spike-reactive B cells are detected at 9 months

Authors

Jeffrey Seow, Carl Graham, Sadie R. Hallett, ..., Julie Fox, Michael H. Malim, Katie J. Doores

Correspondence

katie.doores@kcl.ac.uk

In brief

Seow et al. isolate antibodies following double vaccination with AZD1222. Isolated antibodies target epitopes overlapping with the epitopes of infection-derived antibodies and show potent neutralization of SARS-CoV-2 variants of concern (VOCs), including Omicron. Despite low serum neutralization at 9 months post-vaccine, Spike-reactive B cells are detected.

Seow et al., 2022, Cell Reports *39*, 110757
May 3, 2022 © 2022 The Authors.
https://doi.org/10.1016/j.celrep.2022.110757

CellPress

Cell Reports

CellPress
OPEN ACCESS

Article

ChAdOx1 nCoV-19 vaccine elicits monoclonal antibodies with cross-neutralizing activity against SARS-CoV-2 viral variants

Jeffrey Seow,[1,5] Carl Graham,[1,5] Sadie R. Hallett,[1] Thomas Lechmere,[1] Thomas J.A. Maguire,[1] Isabella Huettner,[1] Daniel Cox,[1] Hataf Khan,[1] Suzanne Pickering,[1] Rebekah Roberts,[2] Anele Waters,[2] Christopher C. Ward,[1] Christine Mant,[3] Michael J. Pitcher,[4] Jo Spencer,[4] Julie Fox,[1,2] Michael H. Malim,[1] and Katie J. Doores[1,6,*]

[1]Department of Infectious Diseases, School of Immunology and Microbial Sciences, King's College London, London, UK
[2]Harrison Wing, Guy's and St Thomas' NHS Trust, London, UK
[3]Infectious Diseases Biobank, Department of Infectious Diseases, School of Immunology and Microbial Sciences, King's College London, London, UK
[4]Peter Gorer Department of Immunobiology, School of Immunology and Microbial Sciences, King's College London, London, UK
[5]These authors contributed equally
[6]Lead contact
*Correspondence: katie.doores@kcl.ac.uk
https://doi.org/10.1016/j.celrep.2022.110757

SUMMARY

Although the antibody response to COVID-19 vaccination has been studied extensively at the polyclonal level using immune sera, little has been reported on the antibody response at the monoclonal level. Here, we isolate a panel of 44 anti-SARS-CoV-2 monoclonal antibodies (mAbs) from an individual who received two doses of the ChAdOx1 nCoV-19 (AZD1222) vaccine at a 12-week interval. We show that, despite a relatively low serum neutralization titer, Spike-reactive IgG+ B cells are still detectable 9 months post-boost. Furthermore, mAbs with potent neutralizing activity against the current SARS-CoV-2 variants of concern (Alpha, Gamma, Beta, Delta, and Omicron) are present. The vaccine-elicited neutralizing mAbs form eight distinct competition groups and bind epitopes overlapping with neutralizing mAbs elicited following SARS-CoV-2 infection. AZD1222-elicited mAbs are more mutated than mAbs isolated from convalescent donors 1–2 months post-infection. These findings provide molecular insights into the AZD1222 vaccine-elicited antibody response.

INTRODUCTION

The SARS-CoV-2-encoded Spike glycoprotein is the key target for neutralizing antibodies (nAbs) generated in response to natural infection. The Spike trimer consists of two subunits: S1, which is required for interaction with the ACE2 receptor on target cells, and S2, which orchestrates membrane fusion. Many monoclonal antibodies (mAbs) have been isolated from SARS-CoV-2-infected individuals, allowing identification of key neutralizing epitopes on Spike (Andreano et al., 2021a; Barnes et al., 2020; Brouwer et al., 2020; Graham et al., 2021; Piccoli et al., 2020; Robbiani et al., 2020; Rogers et al., 2020; Seydoux et al., 2020; Tortorici et al., 2020). Neutralizing epitopes are present on the receptor-binding domain (RBD) and the N-terminal domain (NTD) of Spike and S2. RBD-specific nAbs tend to be potently neutralizing and target four epitopes (Barnes et al., 2020; Dejnirattisai et al., 2021; Yuan et al., 2020b), including the receptor-binding motif (RBM), which interacts directly with the ACE2 receptor. Furthermore, several non-overlapping neutralizing epitopes on NTD have been identified that are susceptible to sequence variation in this region (Cerutti et al., 2021; Graham et al., 2021; McCallum et al., 2021; Suryadevara et al., 2021). SARS-CoV-2 infection also generates a large proportion of non-neutralizing antibodies of which the biological function is not fully understood (Anderson et al., 2021; Beaudoin-Bussieres et al., 2022; Li et al., 2021). Combined, studying the antibody response to SARS-CoV-2 infection has generated an antigenic map of the Spike surface (Corti et al., 2021; Dejnirattisai et al., 2021).

Following the emergence of SARS-CoV-2 in the human population, vaccines against COVID-19 have been rapidly developed. Most licenced vaccines use, or encode, a SARS-CoV-2 Spike antigen to elicit both humoral and cellular responses, and many have shown remarkable efficacy in Phase III trials (Baden et al., 2021; Polack et al., 2020; Voysey et al., 2021). However, there are concerns that vaccine efficacy could be reduced against newly emerging SARS-CoV-2 variants of concern (VOCs), in particular against the Alpha (B.1.1.7), Beta (B.1.351), Gamma (P.1), Delta (B.1.617.2) and Omicron (B.1.1.529) variants, which harbor mutations throughout Spike. Serum-neutralizing activity against viral variants has been reported in many double-vaccinated individuals, albeit at a reduced potency (Alter et al., 2021; Collier et al., 2021; Edara et al., 2021; Monin et al.,

This is an open access article under the CC BY license (http://creativecommons.org/licenses/by/4.0/).

2021; Supasa et al., 2021; Wang et al., 2021d; Zhou et al., 2021). Despite this reduction, real-world data show that current COVID-19 vaccines are still highly effective in preventing severe disease and hospitalizations in locations where SARS-CoV-2 VOCs are prevalent (Emary et al., 2021; Lopez Bernal et al., 2021; Madhi et al., 2021).

Whereas the antibody response to COVID-19 vaccination has been studied extensively at the polyclonal level using immune sera (Alter et al., 2021; Collier et al., 2021; Dejnirattisai et al., 2021; Edara et al., 2021; Emary et al., 2021; Monin et al., 2021; Supasa et al., 2021; Wall et al., 2021; Wang et al., 2021d; Zhou et al., 2021), little has been reported on the antibody response at the monoclonal level (Amanat et al., 2021; Andreano et al., 2021b; Cho et al., 2021; Turner et al., 2021; Wang et al., 2021d). To address this paucity of information, we isolated a panel of 44 anti-SARS-CoV-2 monoclonal antibodies (mAbs) from an individual (VA14) who had received two doses of the ChAdOx1 nCoV-19(AZD1222) vaccine at a 12-week interval (Figure 1A). The AZD1222 vaccine is a replication-defective chimpanzee adenovirus-vectored vaccine expressing the full-length Wuhan SARS-CoV-2 spike glycoprotein gene (Ramasamy et al., 2021; Voysey et al., 2021). Even though low serum neutralization titers (ID_{50} ~100) were detected in VA14 at 4 months post-vaccine booster, nAbs were isolated that displayed potent cross-neutralizing activity against SARS-CoV-2 VOCs (IC_{50} values as low as 0.003 μg/mL), including the highly mutated Omicron VOC. The AZD1222 vaccine elicited NTD- and RBD-specific nAbs that bind epitopes overlapping with nAbs generated following natural infection. Assessment at 9 months post-second-vaccine dose revealed the presence of Spike-reactive IgG+ B cells despite undetectable neutralization. These data suggest that, although plasma neutralization may be suboptimal for protection from infection, memory B cells may be sufficient to provide rapid-recall responses to protect from serious illness/hospitalizations upon re-infection.

RESULTS

Serum-neutralizing activity following AZD1222 vaccination

Plasma and peripheral blood mononuclear cells (PBMCs) were isolated from donor VA14 (23 years, white male) at 4 months (time point 1, TP1) and 9 months (time point 2, TP2) after receiving two doses of the AZD1222 vaccine at a 12-week interval (Figure 1A). VA14 reported no previous SARS-CoV-2 infection (based on regular PCR testing), did not have N-specific IgG in their plasma at the time of sampling, and was therefore presumed to be SARS-CoV-2 naive. Presence of IgG to Spike was determined by ELISA (Figure 1B), and a semi-quantitative ELISA measured 0.39 and 0.17 μg/mL of Spike IgG at TP1 and TP2, respectively.

Plasma-neutralizing activity was measured using an HIV-1 (human immunodeficiency virus type 1)-based virus particles, pseudotyped with the Spikes of SARS-CoV-2 VOCs, including AZD1222-matched Spike (Wuhan-1, WT), and VOCs Alpha, Gamma, Beta, and Delta, and a HeLa cell line stably expressing the ACE2 receptor (Graham et al., 2021; Seow et al., 2020). Overall, neutralization titers at 4 months post-vaccine boost (TP1) were low. ID_{50}s of ~100 were measured against WT and Gamma but were reduced against Alpha, Delta, and Beta (Figure 1C). Although weak binding to Spike was observed at TP2, neutralization was not detected at a serum dilution of 1:20 (Figure 1D). Similar neutralization levels were observed against SARS-CoV-2 live virus (England 02/2020/407,073) (Figure 1E).

Spike-reactive B cells detected up to 1 year following AZD1222 vaccination

Next, we determined the percentage of RBD or Spike-reactive IgG-expressing B cells at 4 and 9 months post-vaccine booster using flow cytometry (Figures 1F and S1A–S1D). A total of 0.25% of IgG+ B cells were Spike reactive, and 0.06% were RBD reactive at 4 months post-vaccine booster. Despite the undetectable neutralization by serum antibodies produced by plasma cells at 9 months post-vaccine booster, 0.27% of IgG + B cells were Spike reactive.

AZD1222 vaccination elicits antibodies targeting epitopes on NTD, RBD, S2, and Spike

RBD or Spike-reactive B cells at 4 months post-vaccine booster were sorted into individual wells, and the antibody heavy- and light-chain genes were rescued by reverse transcription followed by nested PCR using gene-specific primers (Graham et al., 2021). Variable regions were ligated into IgG1 heavy- and light-chain expression vectors using Gibson assembly and directly transfected into HEK293T/17 cells. Crude supernatants containing IgG were used to confirm specificity to Spike, and the variable heavy and light chain regions of Spike-reactive mAbs were sequenced. In total, 44 Spike-reactive mAbs were isolated from VA14.

Binding to Spike, S1, RBD, NTD, and S2 was determined by ELISA and used to identify the domain specificity of each mAb (Figure 2A). Of the 40 mAbs isolated using the stabilized Spike-sorting antigen, 45% (18/40) bound RBD, 35% (14/40) bound NTD, 17.5% (7/40) bound S2, and 2.5% (1/40) bound Spike only (Figure 2B). An additional four RBD-specific mAbs were isolated using the RBD-sorting probe. A similar distribution between mAbs targeting RBD, NTD, and S2 was seen for mAbs isolated from convalescent donors 6–8 weeks post-onset of symptoms (POS) (Graham et al., 2021).

AZD1222 vaccination elicits neutralizing and non-neutralizing antibodies against epitopes across the full Spike

Neutralizing activity of mAbs was initially measured using HIV-1 virus particles pseudotyped with SARS-CoV-2 Spike encoded by the AZD1222 vaccine. Twenty six of 44 mAbs (59.1%) displayed neutralizing activity of which 21/26 (80.8%) were RBD-specific, 4/26 (15.5%) were NTD-specific and 1/26 (3.8%) only bound Spike (Figure 2B) consistent with previous studies (Graham et al., 2021; McCallum et al., 2021). None of the S2-specific nAbs showed neutralizing activity. 95.5% of RBD-specific mAbs and 38.6% of NTD-specific mAbs had neutralizing activity (Figure 2B). Neutralization potency against wild-type Spike ranged from 0.01–7.3 μg/mL. As previously reported for natural infection, RBD-specific nAbs had a lower geometric mean IC_{50}

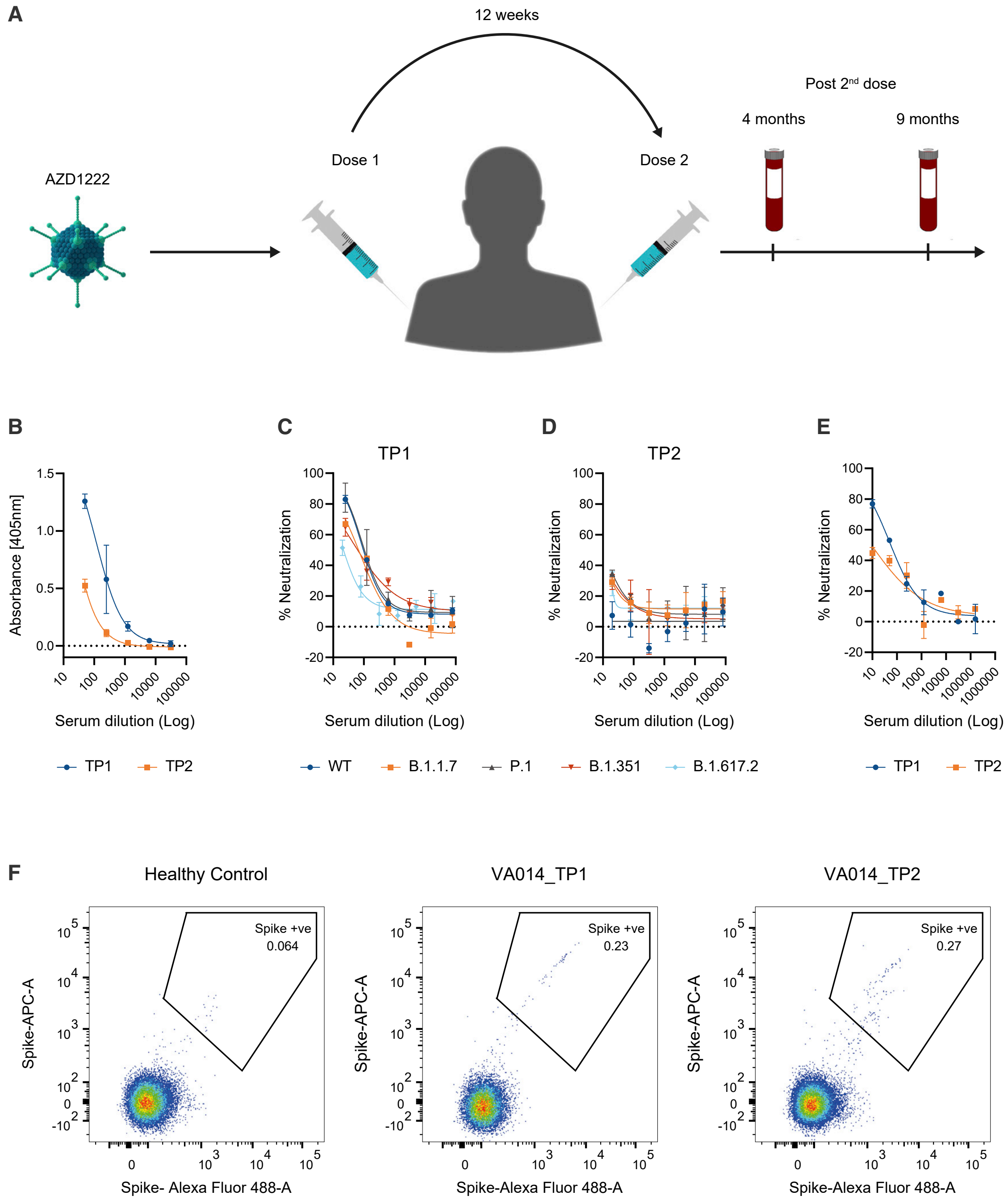

Figure 1. VA14 plasma neutralization and Spike-reactive B cells

(A) Timeline of AZD1222 vaccination, and blood sampling for donor VA14.

(B–D) Plasma IgG binding to Spike at TP1 (4 months post-booster) and TP2 (9 months post-booster). Plasma-neutralizing activity against HIV-1-based virus particles, pseudotyped with the Wuhan, B.1.1.7, P.1, B.1.351, or B.1.617.2 Spike at (C) TP1 and (D) TP2. Experiments were performed in duplicate and repeated twice. A representative dataset is shown. Error bars represent the range of the value for experiments performed in duplicate (not shown when smaller than symbol size).

(E) Plasma-neutralizing activity against neutralization of SARS-CoV-2 (England 02/2020/407,073) at TP1 and TP2. Experiments were performed in duplicate.

(F) Fluorescent-activated cell sorting (FACS) showing percentage of CD19+ IgG+ B cells binding to SARS-CoV-2 Spike at TP1 and TP2. A healthy control PBMC sample collected prior to the COVID-19 pandemic was used to measure background binding to Spike. The full gating strategy and sorting of RBD-specific B cells can be found in Figure S1.

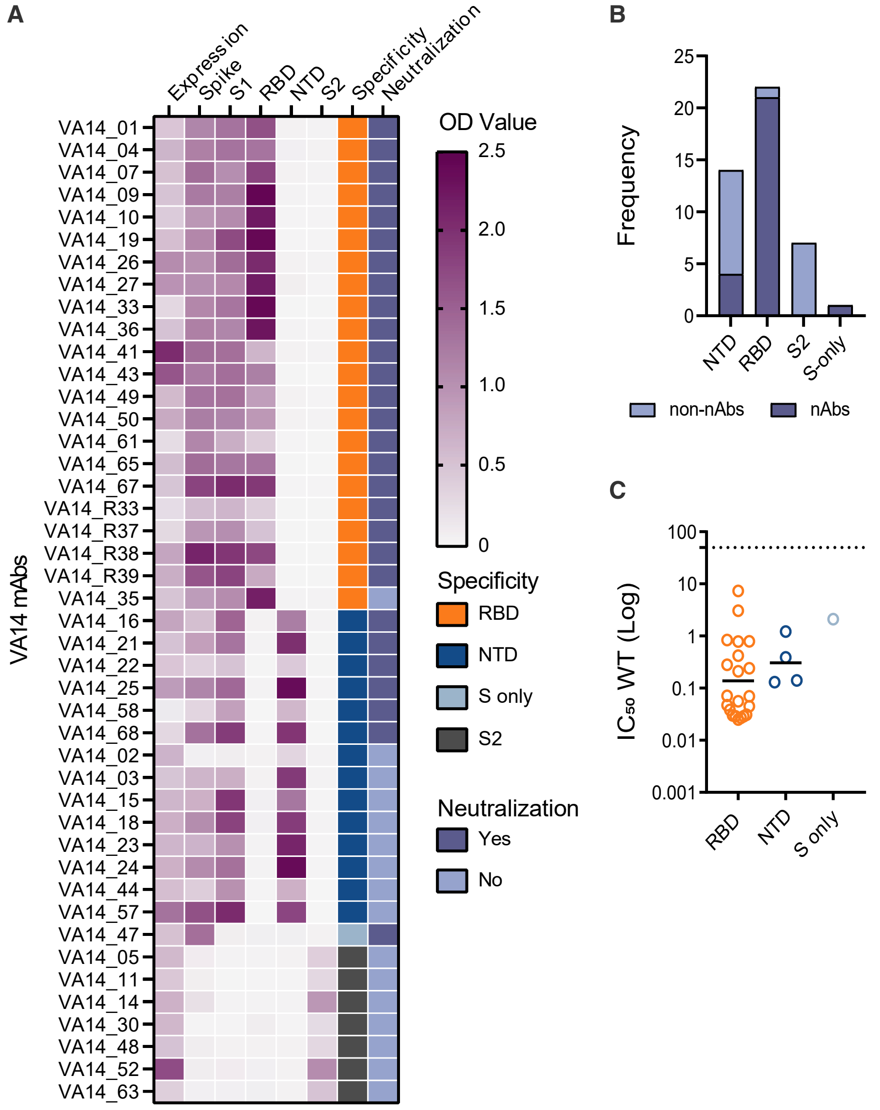

Figure 2. AZD1222 elicits neutralizing and non-neutralizing antibodies targeting RBD, NTD, S1, and S2 domains of Spike

(A) Heatmap showing IgG expression level and binding to SARS-CoV-2 Spike domains, RBD, NTD, S1, and S2. The figure reports OD values from a single experiment (range 0–2.5) for undiluted supernatant from small-scale transfection of 44 cloned mAbs. Antigen binding was considered positive when OD at 405 nm was >0.2 after background was subtracted. SARS-CoV-2 Spike domain specificity for each antibody is indicated. Neutralization activity was measured against wild-type (WT; Wuhan) pseudotyped virus using either small-scale purified IgG or concentrated supernatant.

(B) Frequency of neutralizing and non-neutralizing antibodies targeting RBD, NTD, S-only, or S2. Graph includes only mAbs isolated using Spike as antigen bait for B cell sorting.

(C) Neutralization potency (IC_{50}) against WT (Wuhan) pseudotyped virus for mAbs targeting RBD, NTD, or non-S1. The black line represents the geometric mean IC_{50}. Neutralization experiments were performed in duplicate and carried out at least twice. Related to Table S1.

compared with NTD-specific nAbs (Figure 2C) (Graham et al., 2021; Liu et al., 2020b).

AZD1222-elicited mAbs are more highly mutated than mAbs from natural infection

The heavy and light chain variable regions of Spike-reactive mAbs were sequenced, and the germline usage and level of somatic hypermutation (SHM) were determined using IMGT (Brochet et al., 2008). Averages of 4.9% and 2.8% divergence from V_H and V_L germlines, respectively, were observed at the nucleotide level for AZD1222-elicited mAbs (Figure 3A), which is higher than mAbs isolated in our previous study from convalescent individuals 3–8 weeks post-onset of symptoms (1.9% and 1.4% for V_H and V_L, respectively) (Graham et al., 2021). Three pairs of related clones were identified (Figure S2A).

Germline gene usage and divergence from germline of both neutralizing and non-neutralizing AZD1222 mAbs were compared with a database of SARS-CoV-2-specific mAbs isolated from convalescent individuals (n = 1,292) (Raybould et al., 2021) as well as paired heavy and light chains of IgG B cell receptors (BCRs) from blood of CD19+ B cells from healthy individuals, representative of circulating IgG-expressing B cell repertoire (n = 862) (Siu et al., 2022). Since the SARS-CoV-2 mAb database included amino acid sequences for only some mAbs, divergence from germline was determined at the amino acid level (which correlated well with nucleotide divergence [Figure S2B]). AZD1222-elicited mAbs from donor VA14 had a statistically higher amino acid mutation (V_H 9.2% and V_L 6.1%) compared with mAbs isolated from SARS-CoV-2 convalescent donors (V_H 4.2% and V_L 3.0%) but had a level similar to that of B cell receptors from healthy subjects (V_H 10.9% and V_L 8.0%) (Figures 3B and 3C). Similar differences in mutation levels were observed for both neutralizing and non-neutralizing antibodies (Figure S2C).

An enrichment in VH3-30 and VH3-53 germline gene usage was observed for both SARS-CoV-2 infection and AZD1222-elicited mAbs, similar to that seen for COVID-19 mRNA vaccine-elicited mAbs (Wang et al., 2021d) (Figure 3D). Three of 21 RBD-specific nAbs used the VH3-53/3-66 germlines that are common among nAbs that directly bind the ACE2-binding site on Spike (Barnes et al., 2020; Graham et al., 2021; Kim et al., 2021; Robbiani et al., 2020; Yuan et al., 2020c). An enrichment of VH3-15, VH3-48, VH4-34, and VH4-59 germline use was observed for AZD1222-elicited mAbs compared with mAbs isolated from

CellPress
OPEN ACCESS

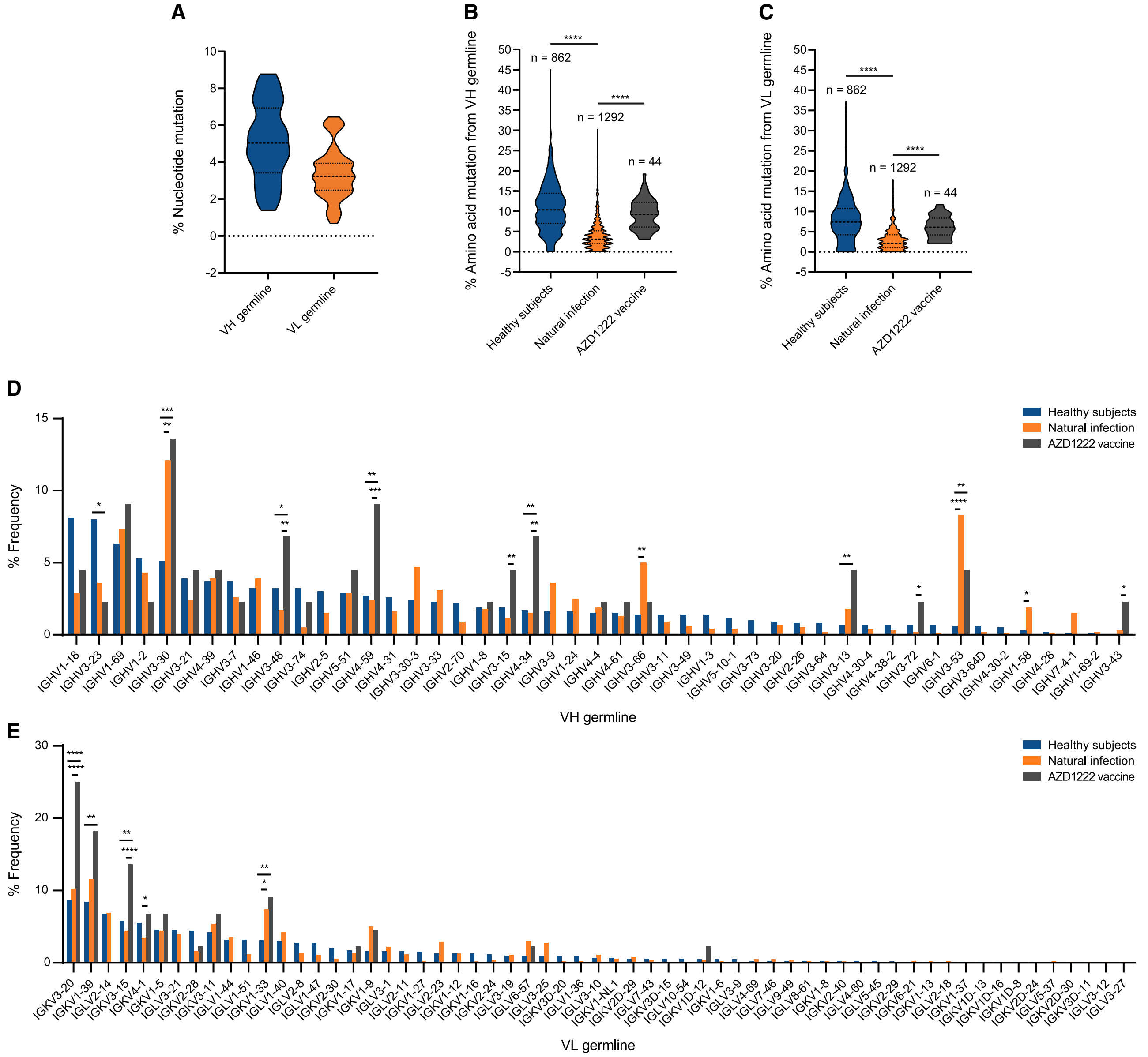

Figure 3. AZD1222-elicited monoclonal antibodies are more mutated than those elicited following SARS-CoV-2 infection

(A–C) Truncated violin plot showing the percentage of nucleotide mutation compared with germline for the VH and VL genes of Spike-reactive mAbs isolated following AZD1222. Divergence from germline (based on amino acid alignments) for (B) VH and (C) VL genes for Spike-reactive mAbs from natural infection, AZD1222 vaccination, and IgG BCRs from SARS-CoV-2-naive individuals (Siu et al., 2022). D'Agostino and Pearson tests were performed to determine normality. Based on the result, a Kruskal-Wallis test with Dunn's multiple comparison post hoc test was performed. *p < 0.0332, **p < 0.0021, ***p < 0.0002, and ****<0.0001.
(D and E) Graph showing the relative abundance of (D) VH and (E) VL genes in mAbs elicited from AZD1222 vaccination compared with SARS-CoV-2 infection mAbs (Raybould et al., 2021) and IgG BCRs from SARS-CoV-2-naive individuals (Siu et al., 2022). A two-sided binomial test was used to compare the frequency distributions. *p < 0.0332, **p < 0.0021, ***p < 0.0002, and ****<0.0001. Related to Figure S2.

convalescent donors; 11/44 (25.0%) and 8/44 (18.2%) mAbs used VK3-20 and VK1-39 light chains, respectively (Figure 3E).

AZD1222-elicited nAbs bind epitopes overlapping with nAbs generated in response to SARS-CoV-2 infection

To gain insight into the epitopes targeted by the AZD1222-elicited nAbs, competition ELISAs with trimeric Spike and previously characterized nAbs isolated from SARS-CoV-2-infected individuals were performed. The panel of competing antibodies encompassed four RBD-, two NTD-, and one Spike-only competition groups (Graham et al., 2021) (Figures 4A and 4B). Additionally, the ability of nAbs to inhibit the interaction between Spike and the ACE2 receptor was determined by flow cytometry (Figure 4D).

Four RBD-neutralizing antibody classes have been previously identified and characterized (Figure 4E) (Barnes et al., 2020;

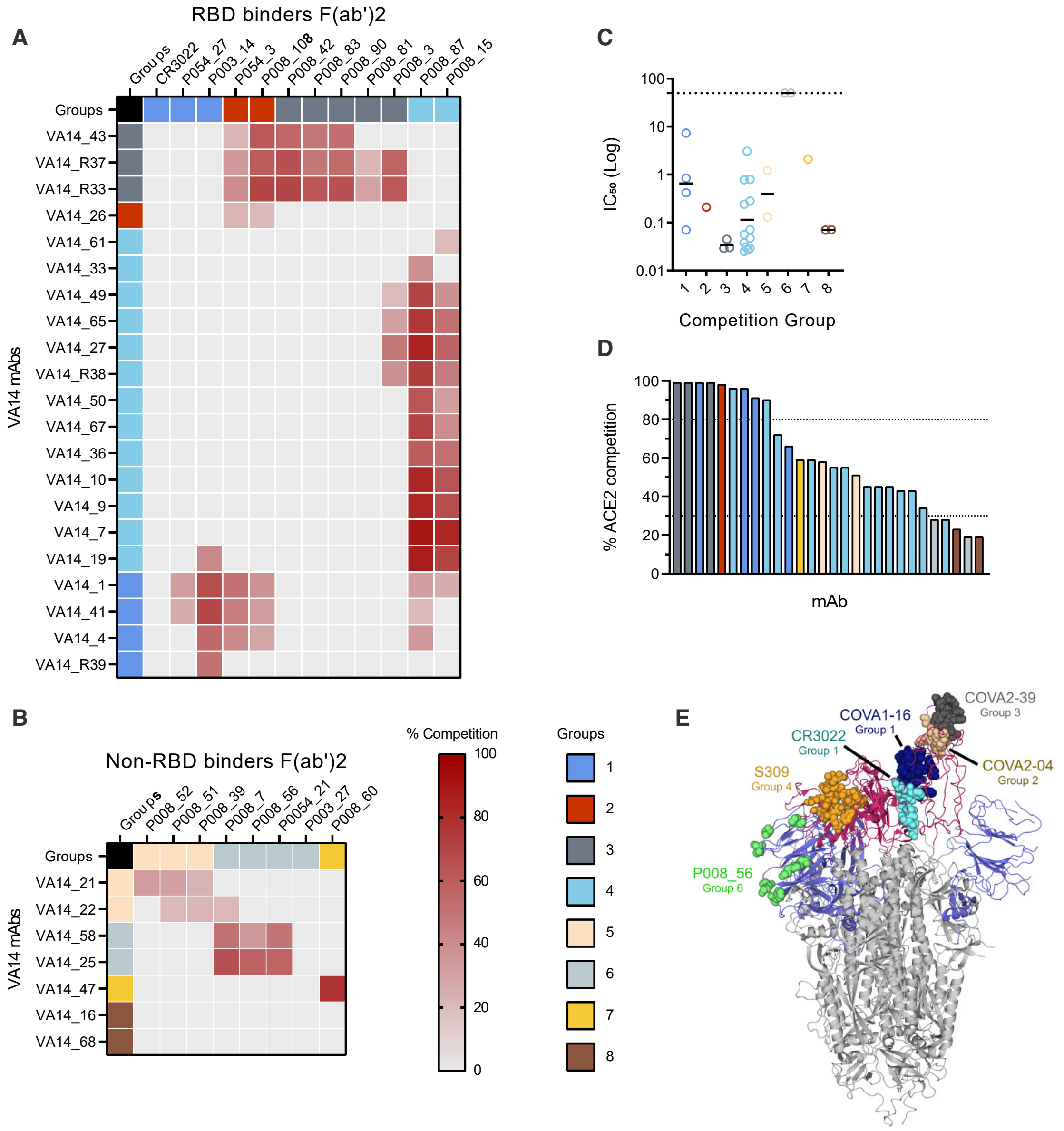

Figure 4. AZD1222 nAbs target epitopes overlapping with nAbs elicited following natural SARS-CoV-2 infection

(A and B) Competitive binding of AZD1222 and SARS-CoV-2 infection-elicited nAbs. Inhibition of IgG binding to SARS-CoV-2 Spike by F(ab)$_2$' fragments was measured. The percentage of competition was calculated using the reduction in IgG binding in the presence of F(ab')$_2$ (at 100 molar excess of the IC_{80}) as a percentage of the maximum IgG binding in the absence of F(ab')$_2$. Competition was measured between (A) RBD-specific and (B) NTD-specific/S-only nAbs. Competition groups were determined according to binding epitopes. Experiments were performed in duplicate. Competition <25% is in white.

(C) Neutralization potency (IC_{50}) of mAbs targeting RBD, NTD, or non-S1 and/or in competition groups 1–8 against SARS-CoV-2 WT pseudotyped virus. Competition groups are color coded according to the key. The black lines represent the geometric mean IC_{50} for each group. Neutralization experiments were performed in duplicate and carried out at least twice.

(D) Ability of nAbs to inhibit the interaction between cell surface ACE2 and soluble SARS-CoV-2 Spike. nAbs (at 600 nM) were pre-incubated with fluorescently labeled Spike before addition to HeLa-ACE2 cells. The percentage reduction in mean fluorescence intensity is reported. Experiments were performed in duplicate. Bars are color coded based on the antibody competition group.

(E) Mapping of previously determined neutralizing and non-neutralizing epitopes on SARS-CoV-2 Spike (PBD: 6XM0) (Zhou et al., 2020). Cartoon representation of Spike showing antibody-binding footprint for nAbs used in competition ELISAs as colored spheres. Epitopes were previously determined using crystal structures or cryo-electron microscopy of RBD or Spike-Fab complexes; COVA2-04 (gold, group 2 [RBD Class 1], [(PBD: 7JMO] [Wu et al., 2020]), COVA2-39 (gray, group 3 [RBD Class 2] [PBD: 7JMP] [Wu et al., 2020]), S309 (orange, group 4 [RBD Class 3] [PBD: 6WPS] [Pinto et al., 2020]), COVA1-16 and CR3022 (dark blue [PBD: 7JMW] [Liu et al., 2020a] and turquoise [PBD: 6W41] [Yuan et al., 2020c], respectively, group 1 [RBD Class 4]), and P008_056 (green, NTD group 6 [Rosa et al., 2021]). Structures were generated in Pymol using the referenced PBDs.

CellPress
OPEN ACCESS

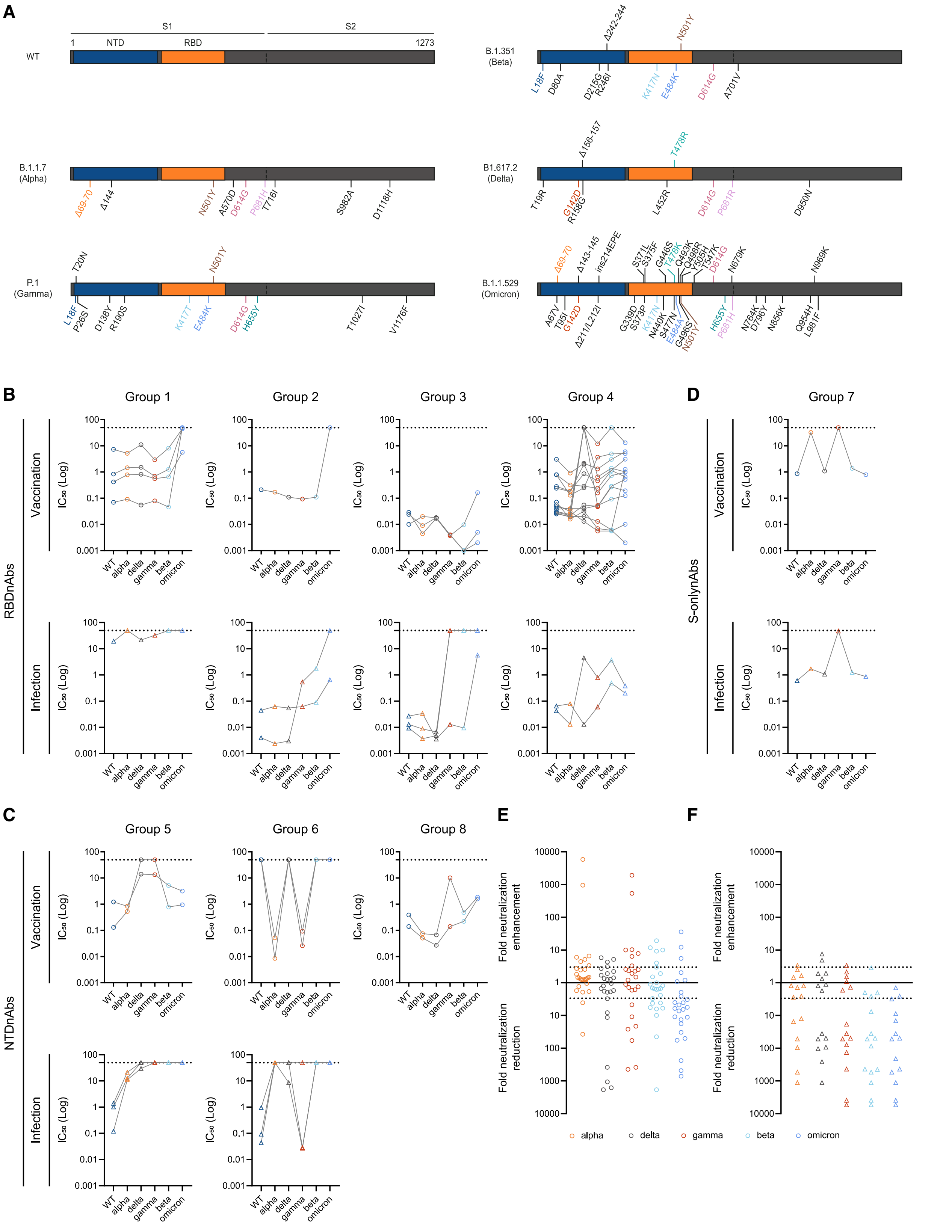

(legend on next page)

Yuan et al., 2020b). nAbs that neutralize by binding to the receptor-binding motif (RBM) (equivalent to RBD class 1) (Barnes et al., 2020; Dejnirattisai et al., 2021; Yuan et al., 2020a) commonly use the VH3-53 or VH3-66 germ lines. As expected, the three VH3-53/VH3-66 VA14 nAbs competed with the group 3 (RBD class 1) infection nAbs as well as competing strongly for ACE2 binding (Figure 4D). Group 3 nAbs were most potent at neutralizing the matched vaccine strain (Wuhan-1) (Figure 4C).

The majority of RBD-specific nAbs isolated from VA14 (13/20) competed with the group 4 (RBD class 3) RBD infection nAbs (Figure 4A) and included both potent and modest neutralizing Abs with varying degrees of ACE2 competition (Figures 4C and 4D). Five VA14 nAbs competed with group 1 (RBD class 4) RBD infection nAbs and showed a wide range of neutralization potencies and levels of ACE2 competition. Only one VA14 nAb (VA14_26) competed with group 2 (RBD class 2) RBD infection nAbs, which also competed strongly with ACE2.

NTD mAbs formed three competition groups (Figure 4B). Non-neutralizing mAbs VA14_25 and VA14_58 competed with NTD group 6 nAbs including P008_056, which has been shown to bind NTD adjacent to the β-sandwich fold (Figure 4E) (Rosa et al., 2021). These two nAbs did not inhibit Spike binding to ACE2 (Figure 4D). nAbs VA14_21 and VA14_22 competed with NTD group 5 nAbs and showed 51%–58% inhibition of Spike binding to ACE2. Two NTD nAbs (group 8) did not compete with any of the infection NTD-specific nAbs or prevent ACE2 binding.

The S-only-binding nAb VA14_47 competed with P008_060 (group 7) (Figure 4B), the only other S-only infection nAb, and showed 59% inhibition of Spike binding to ACE2 (Figure 4D). P008_060 has been shown to bind a neutralizing epitope on the SD1 domain of Spike (manuscript in preparation).

AZD1222-elicited nAbs cross-neutralize SARS-CoV-2 variants of concern

Assessing the cross-neutralizing activity of nAbs isolated from SARS-CoV-2 convalescent donors has revealed that Spike mutations in VOCs selectively hinder neutralizing activity of specific nAb classes (Graham et al., 2021; Wang et al., 2021a, 2021b, 2021c; Wibmer et al., 2021). Therefore, we measured the neutralization potency of AZD1222-elicited nAbs against SARS-CoV-2 variants of concern, including B.1.1.7 (Alpha), B.1.351 (Beta), B.1.617.2 (Delta), P.1 (Gamma), and B.1.1.529 (Omicron) and compared this with nAbs isolated following natural infection (Graham et al., 2021). Spike proteins from these VOCs encode mutations in RBD, NTD, and S2 (Figure 5A). Some RBD mutations are shared among multiple variants; e.g., Alpha, Gamma, Beta, and Omicron all share an N501Y mutation, and Gamma, Beta, and Omicron share a mutation at K417 and E484. In contrast, NTD mutations vary considerably among VOCs and include amino acid mutations as well as insertions and deletions. Omicron encodes >30 mutations in Spike and has been reported to evade neutralization by sera from individuals receiving two doses of a COVID-19 vaccine (Cele et al., 2021; Garcia-Beltran et al., 2022; Gruell et al., 2022; Wu et al., 2022) as well as many SARS-CoV-2-specific monoclonal antibodies (Cameroni et al., 2021; Cao et al., 2021; Planas et al., 2021; VanBlargan et al., 2022). Although a reduction in neutralization potency was observed for some AZD1222 nAbs, RBD- and NTD-specific nAbs with potent cross-neutralization against all VOCs, including Omicron, were identified (Figures 5B and 5C).

All group 3, several group 4 (including VA14_33, VA14_36, VA14R_38 and VA14_61), and one group 1 (VA14R_39) RBD-specific nAbs potently neutralized WT, Alpha, Beta, Gamma, and Delta variants at IC_{50}s below 0.09 μg/mL (Figure 5B), and all but two of these nAbs also neutralized Omicron at IC_{50}s below 0.16 μg/mL. VA14R_37 was most potent against Omicron, neutralizing with an IC_{50} of 0.002 μg/mL. Several nAbs showed enhanced neutralization of VOCs compared with WT (Figure 5E). When nAbs elicited following infection (Graham et al., 2021) and vaccination were compared, infection nAbs showed a greater sensitivity to Spike mutations in VOCs (Figure 5F). Cross-neutralization of nAbs in RBD groups 1, 2, and 3 was observed for AZD1222 nAbs, whereas some infection nAbs in these competition groups showed greatly reduced neutralization of VOCs Gamma, Beta, and Omicron, which share RBD mutations at positions K417, E484, and N501Y (Figure 5A).

RBD group 4 mAbs varied in their neutralization of VOCs. Six of 13 nAbs showed cross-neutralizing activity. The remaining seven showed a >3-fold reduction in neutralization against at least one VOC, with neutralizing activity against Beta, Delta, and Omicron being most greatly reduced. Despite some RBD nAbs showing a decreased neutralization against VOCs, binding to variant RBD in ELISA was retained for most nAbs except group 4 nAbs VA14_19 and VA14_50 (Figure S3A), indicating that binding to RBD does not always correlate with neutralization activity.

Considering the geometric mean IC_{50} values, VA14 NTD-specific nAbs were most potent at neutralizing the Alpha VOC. However, the three NTD-competition groups showed differential sensitivities toward the other four SARS-CoV-2 variants (Figure 5C). For example, group 5 VA14 NTD nAbs had either reduced or lacked neutralization toward Gamma and Delta, whereas group 8 NTD nAbs VA14_16 and VA14_68 maintained neutralizing

Figure 5. AZD1222 generates nAbs with cross-neutralizing activity against SARS-CoV-2 viral variants

(A) Schematic showing mutations present in the Spike of SARS-CoV-2 viral variants of concern (B.1.1.7 [Alpha]), P.1 [Gamma], B.1.351 [Beta], B.1.617.2 [Delta], and B.1.1.529 [Omicron]).

(B) Neutralization by RBD-specific nAbs isolated following AZD1222 vaccination or SARS-CoV-2 infection against main variants of concern. nAbs are separated by competition group (groups 1–4).

(C) Neutralization by NTD-specific nAbs isolated following AZD1222 vaccination or SARS-CoV-2 infection against main variants of concern. nAbs are separated by competition group (groups 5, 6, and 8).

(D) Neutralization by S-only-specific nAbs isolated following AZD1222 vaccination or SARS-CoV-2 infection against main variants of concern. Neutralization experiments were performed in duplicate and carried out at least twice.

(E and F) Fold enhancement or reduction in neutralization IC_{50} against VOCs Alpha, Gamma, Beta, Delta, and Omicron compared with the IC_{50} against WT for (E) AZD1222-elicited mAbs and (F) infection mAbs (Graham et al., 2021). The dotted line indicates a 3-fold reduction or enhancement in neutralization. Related to Figures S3 and S4, and Table S1.

CellPress
OPEN ACCESS

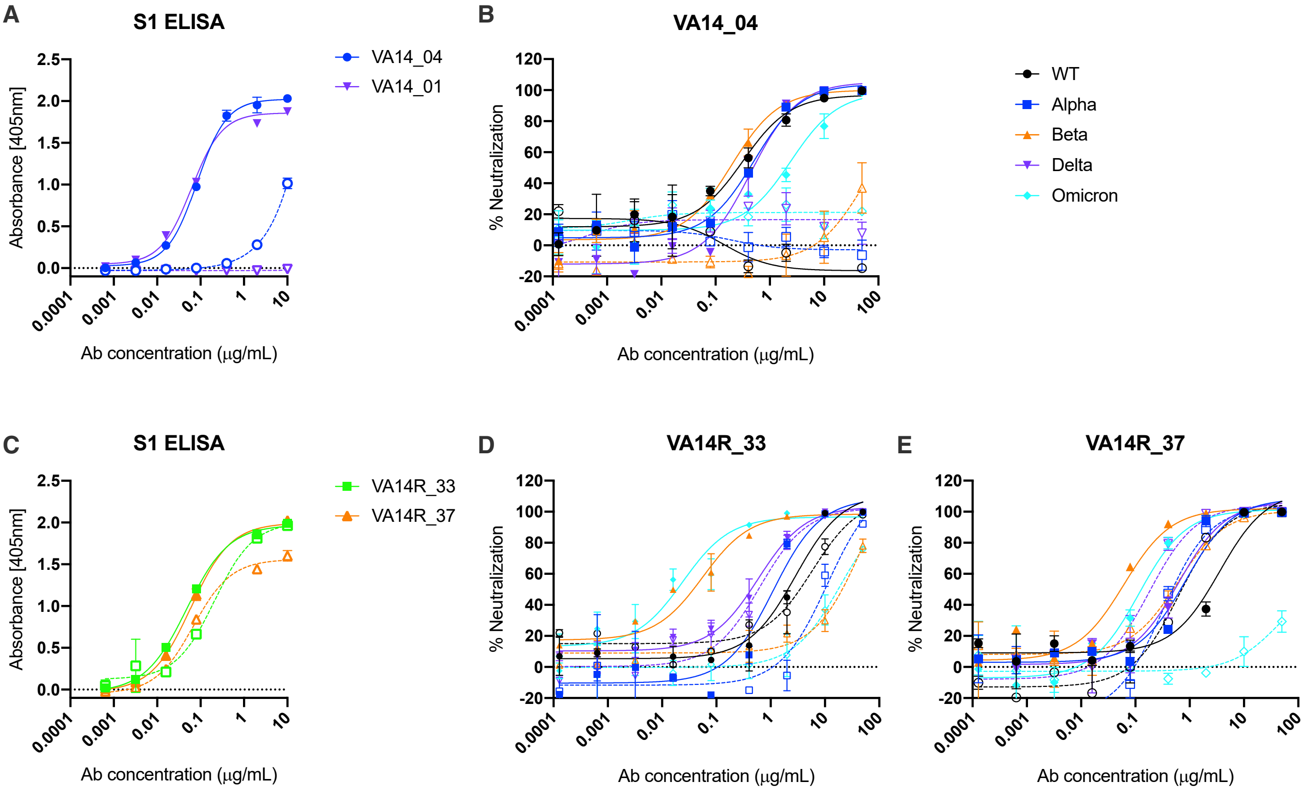

Figure 6. Neutralization activity of reverted germline antibodies

(A) Binding of group 1 reverted mAbs to WT S1 by ELISA. Reverted mAbs (VA14_01_rev and VA14_04 rev) are shown in open symbols and dotted lines.
(B) Comparison of neutralization activity for VA14_04 and germline reverted mAb against WT, Alpha, Beta, Delta, and Omicron. The reverted mAb is shown with open symbols and dotted line.
(C) Binding of group 3 reverted mAbs to WT Spike by ELISA. Reverted mAbs (VA14R_33_rev and VA14R_37 rev) are shown in open symbols and dotted lines.
(D) Neutralization of VA14R_33 and reverted mAb against WT, Alpha, Beta, Delta, and Omicron. The reverted mAb is shown with open symbols and dotted line.
(E) Comparison of neutralization of VA14R_37 and germline reverted mAb against WT, Alpha, Beta, Delta, and Omicron. The reverted mAb is shown with open symbols and dotted line. VOCs are color coded according to the key. Experiments were performed in duplicate and repeated twice. A representative dataset is shown. Error bars represent the range of the value for experiments performed in duplicate (not shown when smaller than symbol size). Related to Figure S5.

activity against all variants. The NTD-specific nAb VA14_16 potently neutralized Gamma, Alpha, Delta, and Beta with an IC_{50} < 0.22 μg/mL and Omicron with an IC_{50} 1.59 μg/mL, and is the only cross-neutralizing NTD-specific nAbs reported thus far (McCallum et al., 2021). Interestingly, two group 6 NTD-specific mAbs that had shown no neutralizing activity against WT pseudotyped virus neutralized both Alpha and Gamma VOCs (Figure 5C). Despite the lack of neutralizing activity, VA14_21 maintained binding to S1 of Gamma, Alpha, Beta, and Delta (Figure S3B). Since the differences in neutralization of VOCs by group 6 NTD-specific nAbs are not reflected in their binding to S1 by ELISA, these data indicate that NTD binding alone is not sufficient for neutralization by this class of mAbs. We have previously shown that Spike binding of the group 6 infection mAb P008_056 is dependent on the heme metabolite biliverdin, and although P008_056 does not neutralize WT pseudovirus, it can potently neutralize live virus (Rosa et al., 2021). Similarly to P008_056, VA14_21 and VA14_61 potently neutralized SARS-CoV-2 live virus (Figure S4A) and binding to Spike was inhibited by biliverdin (Figure S4B), strongly suggesting that these mAbs bind the NTD-neutralizing epitope adjacent to the β-sandwich fold (Rosa et al., 2021).

The S-only-reactive nAb elicited by vaccination (VA14_47) weakly neutralized all SARS-CoV-2 variants except Gamma (Figure 5D), whereas infection mAb P008_060 had modest neutralization against all variants.

Overall, AZD1222 vaccine-elicited nAbs showed greater resistance to Spike mutations in VOCs compared with infection-elicited nAbs (Figure 5E).

Role of somatic hypermutation in neutralization breadth

To probe the role of increasing somatic hypermutation in neutralization breadth, we selected four RBD-specific nAbs and expressed the reverted germline versions. Group 1 nAbs VA14_01 and VA014_04 both use VH3-13 (5.6% and 0.2% divergent from germline, respectively) and VK1-39 (1.4% and 6.4% divergent from germline, respectively) (Figures S5A and S5B). The reverted germline of VA014_01 did not bind to WT Spike in ELISA (Figure 6A). In contrast, VA014_04 reverted germline bound very weakly to WT Spike but did not neutralize WT or any VOCs, thus

demonstrating the importance of SHM for antigen recognition and neutralization for these group 1 mAbs (Figure 6B).

Germline-reverted versions of two group 3 mAbs, VA14R_33 and VA14R_37, were also generated (Figures S5C and S5D). VA14R_33 is encoded by VH3-66 (8.4% mutated) and VK1-33 (3.9% mutated), and VA14R_37 is encoded by VH3-53 (2.4% mutated) and VK3-20 (6.0% mutated). VH3-53/VH3-66 are commonly used by RBM-targeted nAbs, and these germlines have been reported to have amino acid motifs that are pre-configured to recognize RBM (Clark et al., 2021; Yuan et al., 2020a), in particular Asn32-Tyr33 and Ser53-Gly54-Gly55-Ser56. The reverted germlines of both VA14R_33 and VA14_37 retained binding to WT Spike (Figure 6C). Reversion of VA14R_33 to germline reduced the neutralization potency against Alpha, Beta and Omicron (Figure 6D), but neutralization of WT and Delta was largely unaffected. The reverted germline of VA14R_37 was unable to neutralize Omicron (Figure 6E) and had reduced potency against Beta. Interestingly, neutralization potency of germline-reverted VA14R_37 was increased against WT and Delta. Overall, these results highlight the importance of SHM for neutralization breadth and potency against VOCs.

DISCUSSION

Efficacy of COVID-19 vaccines in the face of SARS-CoV-2 emerging viral variants will be critical for control of the current pandemic. Here, we studied the antibody response to two doses of the AZD1222 vaccine administered with a 12-week interval at the monoclonal antibody level. The majority of studies examining immune sera from AZD1222-vaccinated individuals have revealed a lower potency against Alpha (range 2.2- to 9.0-fold) (Dejnirattisai et al., 2021; Emary et al., 2021; Wall et al., 2021), Gamma (2.9-fold) (Dejnirattisai et al., 2021), Beta (range 4.0- to 9.0-fold) (Dejnirattisai et al., 2021; Madhi et al., 2021; Zhou et al., 2021) and Delta (range 4.3- to 9.0-fold) (Liu et al., 2021; Wall et al., 2021) compared with neutralization of Wuhan or D614G variants, and very limited neutralization against Omicron (Wu et al., 2022). Although VA14 had a low plasma-neutralizing activity (ID_{50} ~1:100) at 4 months post-vaccine booster, 59.1% of Spike-reactive mAbs isolated from antigen-reactive B cells had neutralizing activity against the matched vaccine strain, and many of these mAbs displayed potent cross-neutralizing activity against current SARS-CoV-2 VOCs. Similar to previous studies, RBD and NTD were the predominant targets for neutralizing antibodies (80.8% and 15.5% of nAbs, respectively) (Graham et al., 2021; McCallum et al., 2021). Importantly, we identified RBD-specific nAbs from competition groups 1, 3, and 4, as well as NTD-specific nAbs, that cross-neutralized all five VOCs, including the highly divergent Omicron. Therefore, the polyclonal nature of the nAb response elicited by AZD1222 vaccination will likely help limit full vaccine escape in the face of emerging Spike mutations.

Competition ELISAs revealed that nAbs elicited by AZD1222 vaccination target overlapping epitopes of nAbs elicited from natural SARS-CoV-2 infection. However, despite similar antibody footprints, vaccine-elicited nAbs from RBD competition groups 1 to 4 showed greater neutralization breadth than those elicited from natural infection. This was also apparent for some NTD-specific nAbs. This increased neutralization breadth is likely due to the increased divergence from germline in AZD1222-elicited nAbs (isolated 4 months post-booster) compared with nAbs isolated following natural infection (isolated 2–8 weeks post-onset of symptoms) leading to better tolerance of Spike mutations in VOCs. Somatic hypermutation was shown to be critical for antigen recognition by group 1 RBD mAbs VA14_01 and VA14_04, whose reverted germlines had low or undetectable Spike binding by ELISA and lacked neutralization activity. In contrast, the reverted germline of VA14R_37 and VA14R_33 retained Spike reactivity but had a reduced neutralization breadth against Omicron and Beta VOCs. Previous structural analysis of RBM nAbs CC12.1 and CC12.3 (also encoded by VH3-53) revealed that germline encoded amino acid motifs Asn32-Tyr33 and Ser53-Gly54-Gly55-Ser56 are critical for antigen recognition (Yuan et al., 2020a). A germline-reverted version RBM mAb CV30, also encoded by VH3-53, retained Spike-binding activity but had reduced neutralization potency (Hurlburt et al., 2020). The observation that mutation of the VA14R_37 germline VH and VK genes leads to potent neutralization of Beta and Omicron variants demonstrates the importance of somatic hypermutation for enhancing neutralization breadth. Indeed, several other studies have shown that increased somatic hypermutation enhances neutralization breadth against VOCs (Chen et al., 2021; de Mattos Barbosa et al., 2021; Gaebler et al., 2021; Goel et al., 2021; Muecksch et al., 2021). Analysis of the antibody-antigen interaction at the molecular level will give further insight into the specific mechanisms of increased neutralization breadth for AZD1222-elicited nAbs.

Although Spike-reactive mAbs generated following AZD1222 have not previously been reported, several studies report mAbs isolated following mRNA COVID-19 vaccination (Amanat et al., 2021; Andreano et al., 2021b; Cho et al., 2021; Turner et al., 2021; Wang et al., 2021d). Comparison between epitopes targeted by mRNA- and AZD1222-elicited nAbs showed a higher proportion of RBM-targeted nAbs following mRNA vaccination (Gaebler et al., 2021; Hurlburt et al., 2020). A similar enrichment in VH3-53 and VH3-30 germline usage was also observed (Andreano et al., 2021b; Wang et al., 2021d). Despite differences in the timing of mAb isolation across reported studies, the AZD1222 mAbs identified had a higher level of SHM compared with mRNA-elicited mAbs and showed greater cross-neutralizing activity (Scheid et al., 2009; Seydoux et al., 2020). Possible reasons for these differences include (1) timing of mAb isolation following vaccine booster, (2) timing of vaccine boosters (3 weeks for mRNA studies versus 12 weeks in this study), (3) a prolonged antigen persistence for ChAdOx1 vectored Spike (which may also be relevant to the Ad26 vectored Ad26.COV2.S vaccine [Sadoff et al., 2021]), or (4) differences in Spike antigen encoded by each vaccine (in particular, mRNA-1273 [Moderna] and BNT162b2 [Pfizer] vaccines encode Spike with stabilizing mutations and a mutation that prevents S1/S2 cleavage [Jackson et al., 2020; Walsh et al., 2020]). Understanding these factors will be important for optimizing vaccine strategies aimed at eliciting the broadest nAb response against both known and newly emerging VOCs.

Plasma was not available to determine the peak neutralizing response in VA14 and therefore the relative decline in

neutralization following AZD1222 vaccination. The neutralizing antibody titer was low 4 months post-vaccine boost, and it is not known whether this level would be sufficient to provide sterilizing or near-sterilizing immunity. However, the identification of B cells producing antibodies with potent cross-neutralizing activity against non-overlapping epitopes and the presence of Spike+ IgG+ B cells at ~1 year post-vaccine prime suggests that a rapid recall response will likely occur, which could be sufficient to protect against severe disease and/or hospitalization in the face of VOCs.

In summary, we show that AZD1222 vaccine administered at a 12-week interval can elicit nAbs with potent cross-neutralizing activity against current SARS-CoV-2 VOCs, including Omicron, that target non-overlapping epitopes on RBD and NTD. Despite undetectable plasma-neutralizing activity, Spike-reactive IgG+ B cells are detected up to 1 year following initial vaccine priming. These data provide important insights into long-term immunity and protection against SARS-CoV-2 emerging viral variants.

Limitations of the study

The main limitation of this study is that it examines mAbs isolated from only one AZD1222-vaccinated individual. How representative these mAbs are of the humoral immune response arising from AZD1222 needs to be investigated further by isolating mAbs from other AZD1222 vaccine recipients. We have not determined how neutralization breadth against VOCs is achieved by the reported mAbs. Further studies examining the epitopes recognized by neutralizing antibodies with broad and potent activity, particularly against Omicron, will be important for optimizing immunogens that elicit mAbs with broad activity. Finally, we have not studied the protective activity of these mAbs *in vivo* or the amount required to achieve sterilizing immunity.

STAR★METHODS

Detailed methods are provided in the online version of this paper and include the following:

- KEY RESOURCES TABLE
- RESOURCE AVAILABILITY
  - Lead contact
  - Materials availability
  - Data and code availability
- EXPERIMENTAL MODEL AND SUBJECT DETAILS
  - Ethics
  - Bacterial strains and cell culture
- METHOD DETAILS
  - Protein expression and purification
  - ELISA (S, RBD, NTD, S2 or S1)
  - Biliverdin competition ELISA
  - Fab/Fc ELISA
  - IgG digestion to generate F(ab')$_2$
  - F(ab')$_2$ and IgG competition ELISA
  - Semi-quantitative ELISA
  - SARS-CoV-2 pseudotyped virus preparation
  - Neutralization assay with SARS-CoV-2 pseudotyped virus
  - Infectious virus strain and propagation
  - Infectious virus neutralization assay
  - Antigen-specific B cell sorting
  - Full-length antibody cloning and expression
  - IgG expression and purification
  - ACE2 competition measured by flow cytometry
  - Monoclonal antibody sequence analysis
- QUANTIFICATION AND STATISTICAL ANALYSIS

SUPPLEMENTAL INFORMATION

Supplemental information can be found online at https://doi.org/10.1016/j.celrep.2022.110757.

ACKNOWLEDGMENTS

We thank Philip Brouwer, Marit van Gils, and Rogier Sanders for the Spike protein construct, Peter Cherepanov for S1 proteins from VOCs, Leo James and Jakub Luptak for the N protein, Wendy Barclay for providing the B.1.617.2 and B.1.1.529 Spike plasmids, and James Voss and Deli Huang for providing the HeLa-ACE2 cells.

This work was funded by Huo Family Foundation Award to M.H.M. and K.J.D.; MRC Genotype-to-Phenotype UK National Virology Consortium ([MR/W005611/1] to M.H.M. and K.J.D.); Fondation Dormeur, Vaduz for funding equipment to K.J.D.; Wellcome Trust Investigator Award ([106223/Z/14/Z] to M.H.M.); and Wellcome Trust Multi-User Equipment Grant ([208354/Z/17/Z] to M.H.M. and K.J.D.). C.G. and S.H. were supported by the MRC-KCL Doctoral Training Partnership in Biomedical Sciences ([MR/N013700/1]). D.C. was supported by a BBSRC CASE in partnership with GlaxoSmithKline ([BB/V509632/1]). This work and the Infectious Diseases Biobank (C.M.) were supported by the Department of Health via a National Institute for Health Research comprehensive Biomedical Research Centre award to Guy's and St Thomas' NHS Foundation Trust in partnership with King's College London and King's College Hospital NHS Foundation Trust. This study is part of the EDCTP2 program supported by the European Union (grant number RIA2020EF-3008 COVAB to K.J.D., J.F., and M.H.M.). The views and opinions of authors expressed herein do not necessarily state or reflect those of EDCTP. This project is supported by a joint initiative between the Botnar Research Centre for Child Health and the European and Developing Countries Clinical Trials Partnership (K.J.D. and J.F.). This research was funded, in whole or in part, by the Wellcome Trust ([106223/Z/14/Z] and [208354/Z/17/Z]). For the purpose of open access, the author has applied a CC BY public copyright license to any Author Accepted Manuscript version arising from this submission.

AUTHOR CONTRIBUTIONS

Conceptualization, K.J.D., J. Seow, and C.G.; Methodology, J. Seow, C.G., S.R.H., T.L., T.J.A.M., I.H., D.C., H.K., and S.P.; Formal analysis, K.J.D., J. Seow, C.G., S.R.H., H.K., I.H., and M.J.P.; Resources, R.R., A.W., C.C.W., C.M., M.J.P., and J. Spencer; Supervision, K.J.D., J.F., M.H.M., and J. Spencer; Writing – original draft, K.J.D., J. Seow, and C.G. All authors reviewed and edited the manuscript.

DECLARATION OF INTERESTS

The authors declare no competing interests.

Received: September 24, 2021
Revised: March 14, 2022
Accepted: April 6, 2022
Published: May 3, 2022

REFERENCES

Alter, G., Yu, J., Liu, J., Chandrashekar, A., Borducchi, E.N., Tostanoski, L.H., McMahan, K., Jacob-Dolan, C., Martinez, D.R., Chang, A., et al. (2021).

Immunogenicity of Ad26.COV2.S vaccine against SARS-CoV-2 variants in humans. Nature *596*, 268–272.

Amanat, F., Thapa, M., Lei, T., Ahmed, S.M.S., Adelsberg, D.C., Carreno, J.M., Strohmeier, S., Schmitz, A.J., Zafar, S., Zhou, J.Q., et al. (2021). SARS-CoV-2 mRNA vaccination induces functionally diverse antibodies to NTD, RBD, and S2. Cell *184*, 3936–3948.e3910.

Anderson, E.M., Goodwin, E.C., Verma, A., Arevalo, C.P., Bolton, M.J., Weirick, M.E., Gouma, S., McAllister, C.M., Christensen, S.R., Weaver, J., et al. (2021). Seasonal human coronavirus antibodies are boosted upon SARS-CoV-2 infection but not associated with protection. Cell *184*, 1858–1864.e1810.

Andreano, E., Nicastri, E., Paciello, I., Pileri, P., Manganaro, N., Piccini, G., Manenti, A., Pantano, E., Kabanova, A., Troisi, M., et al. (2021a). Extremely potent human monoclonal antibodies from COVID-19 convalescent patients. Cell *184*, 1821–1835.e16.

Andreano, E., Paciello, I., Piccini, G., Manganaro, N., Pileri, P., Hyseni, I., Leonardi, M., Pantano, E., Abbiento, V., Benincasa, L., et al. (2021b). Hybrid immunity improves B cells and antibodies against SARS-CoV-2 variants. Nature *600*, 530–535.

Aricescu, A.R., Lu, W., and Jones, E.Y. (2006). A time- and cost-efficient system for high-level protein production in mammalian cells. Acta Crystallogr. D Biol. Crystallogr. *62*, 1243–1250.

Baden, L.R., El Sahly, H.M., Essink, B., Kotloff, K., Frey, S., Novak, R., Diemert, D., Spector, S.A., Rouphael, N., Creech, C.B., et al. (2021). Efficacy and safety of the mRNA-1273 SARS-CoV-2 vaccine. N. Engl. J. Med. *384*, 403–416.

Barnes, C.O., Jette, C.A., Abernathy, M.E., Dam, K.A., Esswein, S.R., Gristick, H.B., Malyutin, A.G., Sharaf, N.G., Huey-Tubman, K.E., Lee, Y.E., et al. (2020). SARS-CoV-2 neutralizing antibody structures inform therapeutic strategies. Nature *588*, 682–687.

Beaudoin-Bussieres, G., Chen, Y., Ullah, I., Prevost, J., Tolbert, W.D., Symmes, K., Ding, S., Benlarbi, M., Gong, S.Y., Tauzin, A., et al. (2022). A Fc-enhanced NTD-binding non-neutralizing antibody delays virus spread and synergizes with a nAb to protect mice from lethal SARS-CoV-2 infection. Cell Rep. *38*, 110368.

Brochet, X., Lefranc, M.P., and Giudicelli, V. (2008). IMGT/V-QUEST: the highly customized and integrated system for IG and TR standardized V-J and V-D-J sequence analysis. Nucleic Acids Res. *36*, W503–W508.

Brouwer, P.J.M., Caniels, T.G., van der Straten, K., Snitselaar, J.L., Aldon, Y., Bangaru, S., Torres, J.L., Okba, N.M.A., Claireaux, M., Kerster, G., et al. (2020). Potent neutralizing antibodies from COVID-19 patients define multiple targets of vulnerability. Science *369*, 643–650.

Cameroni, E., Bowen, J.E., Rosen, L.E., Saliba, C., Zepeda, S.K., Culap, K., Pinto, D., VanBlargan, L.A., De Marco, A., di Iulio, J., et al. (2021). Broadly neutralizing antibodies overcome SARS-CoV-2 Omicron antigenic shift. Nature *602*, 664–670.

Cao, Y., Wang, J., Jian, F., Xiao, T., Song, W., Yisimayi, A., Huang, W., Li, Q., Wang, P., An, R., et al. (2021). Omicron escapes the majority of existing SARS-CoV-2 neutralizing antibodies. Nature *602*, 657–663.

Carter, M.J., Fish, M., Jennings, A., Doores, K.J., Wellman, P., Seow, J., Acors, S., Graham, C., Timms, E., Kenny, J., et al. (2020). Peripheral immunophenotypes in children with multisystem inflammatory syndrome associated with SARS-CoV-2 infection. Nat. Med. *26*, 1701–1707.

Cele, S., Jackson, L., Khoury, D.S., Khan, K., Moyo-Gwete, T., Tegally, H., San, J.E., Cromer, D., Scheepers, C., Amoako, D.G., et al. (2021). Omicron extensively but incompletely escapes Pfizer BNT162b2 neutralization. Nature *602*, 654–656.

Cerutti, G., Guo, Y., Zhou, T., Gorman, J., Lee, M., Rapp, M., Reddem, E.R., Yu, J., Bahna, F., Bimela, J., et al. (2021). Potent SARS-CoV-2 neutralizing antibodies directed against spike N-terminal domain target a single supersite. Cell Host Microbe *29*, 819–833.e817.

Chen, E.C., Gilchuk, P., Zost, S.J., Suryadevara, N., Winkler, E.S., Cabel, C.R., Binshtein, E., Chen, R.E., Sutton, R.E., Rodriguez, J., et al. (2021). Convergent antibody responses to the SARS-CoV-2 spike protein in convalescent and vaccinated individuals. Cell Rep. *36*, 109604.

Cho, A., Muecksch, F., Schaefer-Babajew, D., Wang, Z., Finkin, S., Gaebler, C., Ramos, V., Cipolla, M., Mendoza, P., Agudelo, M., et al. (2021). Anti-SARS-CoV-2 receptor-binding domain antibody evolution after mRNA vaccination. Nature *600*, 517–522.

Clark, S.A., Clark, L.E., Pan, J., Coscia, A., McKay, L.G.A., Shankar, S., Johnson, R.I., Brusic, V., Choudhary, M.C., Regan, J., et al. (2021). SARS-CoV-2 evolution in an immunocompromised host reveals shared neutralization escape mechanisms. Cell *184*, 2605–2617.e2618.

Collier, D.A., De Marco, A., Ferreira, I., Meng, B., Datir, R., Walls, A.C., Kemp, S.S., Bassi, J., Pinto, D., Fregni, C.S., et al. (2021). Sensitivity of SARS-CoV-2 B.1.1.7 to mRNA vaccine-elicited antibodies. Nature *593*, 136–141.

Corti, D., Purcell, L.A., Snell, G., and Veesler, D. (2021). Tackling COVID-19 with neutralizing monoclonal antibodies. Cell *184*, 3086–3108.

de Mattos Barbosa, M.G., Liu, H., Huynh, D., Shelley, G., Keller, E.T., Emmer, B.T., Sherman, E., Ginsburg, D., Kennedy, A.A., Tai, A.W., et al. (2021). IgV somatic mutation of human anti-SARS-CoV-2 monoclonal antibodies governs neutralization and breadth of reactivity. JCI Insight *6*, e147386.

Dejnirattisai, W., Zhou, D., Ginn, H.M., Duyvesteyn, H.M.E., Supasa, P., Case, J.B., Zhao, Y., Walter, T.S., Mentzer, A.J., Liu, C., et al. (2021). The antigenic anatomy of SARS-CoV-2 receptor binding domain. Cell *184*, 2183–2200.e2122.

Dixon, E.V. (2014). Mechanisms of immunoglobulin deactivation by Streptococcus pyogenes (University of Oxford). PhD Thesis.

Dupont, L., Snell, L.B., Graham, C., Seow, J., Merrick, B., Lechmere, T., Maguire, T.J.A., Hallett, S.R., Pickering, S., Charalampous, T., et al. (2021). Neutralizing antibody activity in convalescent sera from infection in humans with SARS-CoV-2 and variants of concern. Nat. Microbiol. *6*, 1433–1442.

Edara, V.V., Pinsky, B.A., Suthar, M.S., Lai, L., Davis-Gardner, M.E., Floyd, K., Flowers, M.W., Wrammert, J., Hussaini, L., Ciric, C.R., et al. (2021). Infection and vaccine-induced neutralizing-antibody responses to the SARS-CoV-2 B.1.617 variants. N. Engl. J. Med. *385*, 664–666.

Emary, K.R.W., Golubchik, T., Aley, P.K., Ariani, C.V., Angus, B., Bibi, S., Blane, B., Bonsall, D., Cicconi, P., Charlton, S., et al. (2021). Efficacy of ChAdOx1 nCoV-19 (AZD1222) vaccine against SARS-CoV-2 variant of concern 202012/01 (B.1.1.7): an exploratory analysis of a randomised controlled trial. Lancet *397*, 1351–1362.

Gaebler, C., Wang, Z., Lorenzi, J.C.C., Muecksch, F., Finkin, S., Tokuyama, M., Cho, A., Jankovic, M., Schaefer-Babajew, D., Oliveira, T.Y., et al. (2021). Evolution of antibody immunity to SARS-CoV-2. Nature *591*, 639–644.

Garcia-Beltran, W.F., St Denis, K.J., Hoelzemer, A., Lam, E.C., Nitido, A.D., Sheehan, M.L., Berrios, C., Ofoman, O., Chang, C.C., Hauser, B.M., et al. (2022). mRNA-based COVID-19 vaccine boosters induce neutralizing immunity against SARS-CoV-2 Omicron variant. Cell *185*, 457–466.e4.

Goel, R.R., Painter, M.M., Apostolidis, S.A., Mathew, D., Meng, W., Rosenfeld, A.M., Lundgreen, K.A., Reynaldi, A., Khoury, D.S., Pattekar, A., et al. (2021). mRNA vaccination induces durable immune memory to SARS-CoV-2 with continued evolution to variants of concern. Preprint at bioRxiv. https://doi.org/10.1101/2021.08.23.457229.

Graham, C., Seow, J., Huettner, I., Khan, H., Kouphou, N., Acors, S., Winstone, H., Pickering, S., Galao, R.P., Dupont, L., et al. (2021). Neutralization potency of monoclonal antibodies recognizing dominant and subdominant epitopes on SARS-CoV-2 Spike is impacted by the B.1.1.7 variant. Immunity *54*, 1276–1289.e6.

Grehan, K., Ferrara, F., and Temperton, N. (2015). An optimised method for the production of MERS-CoV spike expressing viral pseudotypes. MethodsX *2*, 379–384.

Gruell, H., Vanshylla, K., Tober-Lau, P., Hillus, D., Schommers, P., Lehmann, C., Kurth, F., Sander, L.E., and Klein, F. (2022). mRNA booster immunization elicits potent neutralizing serum activity against the SARS-CoV-2 Omicron variant. Nat. Med. *28*, 477–480.

CellPress
OPEN ACCESS

Gu, Z., Eils, R., and Schlesner, M. (2016). Complex heatmaps reveal patterns and correlations in multidimensional genomic data. Bioinformatics *32*, 2847–2849.

Howarth, M., Liu, W., Puthenveetil, S., Zheng, Y., Marshall, L.F., Schmidt, M.M., Wittrup, K.D., Bawendi, M.G., and Ting, A.Y. (2008). Monovalent, reduced-size quantum dots for imaging receptors on living cells. Nat. Methods *5*, 397–399.

Hurlburt, N.K., Seydoux, E., Wan, Y.H., Edara, V.V., Stuart, A.B., Feng, J., Suthar, M.S., McGuire, A.T., Stamatatos, L., and Pancera, M. (2020). Structural basis for potent neutralization of SARS-CoV-2 and role of antibody affinity maturation. Nat. Commun. *11*, 5413.

Jackson, L.A., Anderson, E.J., Rouphael, N.G., Roberts, P.C., Makhene, M., Coler, R.N., McCullough, M.P., Chappell, J.D., Denison, M.R., Stevens, L.J., et al. (2020). An mRNA vaccine against SARS-CoV-2 - preliminary report. N. Engl. J. Med. *383*, 1920–1931.

Kim, S.I., Noh, J., Kim, S., Choi, Y., Yoo, D.K., Lee, Y., Lee, H., Jung, J., Kang, C.K., Song, K.H., et al. (2021). Stereotypic neutralizing VH antibodies against SARS-CoV-2 spike protein receptor binding domain in patients with COVID-19 and healthy individuals. Sci. Transl Med. *13*, eabd6990.

Lefranc, M.P., Giudicelli, V., Ginestoux, C., Bodmer, J., Muller, W., Bontrop, R., Lemaitre, M., Malik, A., Barbie, V., and Chaume, D. (1999). IMGT, the international ImMunoGeneTics database. Nucleic Acids Res. *27*, 209–212.

Li, D., Edwards, R.J., Manne, K., Martinez, D.R., Schafer, A., Alam, S.M., Wiehe, K., Lu, X., Parks, R., Sutherland, L.L., et al. (2021). In vitro and in vivo functions of SARS-CoV-2 infection-enhancing and neutralizing antibodies. Cell *184*, 4203–4219.e4232.

Liu, C., Ginn, H.M., Dejnirattisai, W., Supasa, P., Wang, B., Tuekprakhon, A., Nutalai, R., Zhou, D., Mentzer, A.J., Zhao, Y., et al. (2021). Reduced neutralization of SARS-CoV-2 B.1.617 by vaccine and convalescent serum. Cell *184*, 4220–4236.e13.

Liu, H., Wu, N.C., Yuan, M., Bangaru, S., Torres, J.L., Caniels, T.G., van Schooten, J., Zhu, X., Lee, C.D., Brouwer, P.J.M., et al. (2020a). Cross-neutralization of a SARS-CoV-2 antibody to a functionally conserved site is mediated by avidity. Immunity *53*, 1272–1280.e1275.

Liu, L., Wang, P., Nair, M.S., Yu, J., Rapp, M., Wang, Q., Luo, Y., Chan, J.F., Sahi, V., Figueroa, A., et al. (2020b). Potent neutralizing antibodies against multiple epitopes on SARS-CoV-2 spike. Nature *584*, 450–456.

Lopez Bernal, J., Andrews, N., Gower, C., Gallagher, E., Simmons, R., Thelwall, S., Stowe, J., Tessier, E., Groves, N., Dabrera, G., et al. (2021). Effectiveness of covid-19 vaccines against the B.1.617.2 (delta) variant. N. Engl. J. Med. *385*, 585–594.

Madhi, S.A., Baillie, V., Cutland, C.L., Voysey, M., Koen, A.L., Fairlie, L., Padayachee, S.D., Dheda, K., Barnabas, S.L., Bhorat, Q.E., et al. (2021). Efficacy of the ChAdOx1 nCoV-19 covid-19 vaccine against the B.1.351 variant. N. Engl. J. Med. *384*, 1885–1898.

McCallum, M., Marco, A., Lempp, F., Tortorici, M.A., Pinto, D., Walls, A.C., Beltramello, M., Chen, A., Liu, Z., Zatta, F., et al. (2021). N-terminal domain antigenic mapping reveals a site of vulnerability for SARS-CoV-2. Cell *184*, 2332–2347.

Monin, L., Laing, A.G., Munoz-Ruiz, M., McKenzie, D.R., Del Molino Del Barrio, I., Alaguthurai, T., Domingo-Vila, C., Hayday, T.S., Graham, C., Seow, J., et al. (2021). Safety and immunogenicity of one versus two doses of the COVID-19 vaccine BNT162b2 for patients with cancer: interim analysis of a prospective observational study. Lancet Oncol. *22*, 765–778.

Muecksch, F., Weisblum, Y., Barnes, C.O., Schmidt, F., Schaefer-Babajew, D., Wang, Z., JC, C.L., Flyak, A.I., DeLaitsch, A.T., Huey-Tubman, K.E., et al. (2021). Affinity maturation of SARS-CoV-2 neutralizing antibodies confers potency, breadth, and resilience to viral escape mutations. Immunity *54*, 1853–1868.e1857.

Piccoli, L., Park, Y.J., Tortorici, M.A., Czudnochowski, N., Walls, A.C., Beltramello, M., Silacci-Fregni, C., Pinto, D., Rosen, L.E., Bowen, J.E., et al. (2020). Mapping neutralizing and immunodominant sites on the SARS-CoV-2 spike receptor-binding domain by structure-guided high-resolution serology. Cell *183*, 1024–1042.e21.

Pickering, S., Betancor, G., Galao, R.P., Merrick, B., Signell, A.W., Wilson, H.D., Kia Ik, M.T., Seow, J., Graham, C., Acors, S., et al. (2020). Comparative assessment of multiple COVID-19 serological technologies supports continued evaluation of point-of-care lateral flow assays in hospital and community healthcare settings. PLoS Pathog. *16*, e1008817.

Pinto, D., Park, Y.J., Beltramello, M., Walls, A.C., Tortorici, M.A., Bianchi, S., Jaconi, S., Culap, K., Zatta, F., De Marco, A., et al. (2020). Cross-neutralization of SARS-CoV-2 by a human monoclonal SARS-CoV antibody. Nature *583*, 290–295.

Planas, D., Saunders, N., Maes, P., Guivel-Benhassine, F., Planchais, C., Buchrieser, J., Bolland, W.H., Porrot, F., Staropoli, I., Lemoine, F., et al. (2021). Considerable escape of SARS-CoV-2 Omicron to antibody neutralization. Nature *602*, 671–675.

Polack, F.P., Thomas, S.J., Kitchin, N., Absalon, J., Gurtman, A., Lockhart, S., Perez, J.L., Perez Marc, G., Moreira, E.D., Zerbini, C., et al. (2020). Safety and efficacy of the BNT162b2 mRNA covid-19 vaccine. N. Engl. J. Med. *383*, 2603–2615.

Ramasamy, M.N., Minassian, A.M., Ewer, K.J., Flaxman, A.L., Folegatti, P.M., Owens, D.R., Voysey, M., Aley, P.K., Angus, B., Babbage, G., et al. (2021). Safety and immunogenicity of ChAdOx1 nCoV-19 vaccine administered in a prime-boost regimen in young and old adults (COV002): a single-blind, randomised, controlled, phase 2/3 trial. Lancet *396*, 1979–1993.

Raybould, M.I.J., Kovaltsuk, A., Marks, C., and Deane, C.M. (2021). CoV-AbDab: the coronavirus antibody database. Bioinformatics *37*, 734–735.

Rees-Spear, C., Muir, L., Griffith, S.A., Heaney, J., Aldon, Y., Snitselaar, J.L., Thomas, P., Graham, C., Seow, J., Lee, N., et al. (2021). The effect of spike mutations on SARS-CoV-2 neutralization. Cell Rep. *34*, 108890.

Robbiani, D.F., Gaebler, C., Muecksch, F., Lorenzi, J.C.C., Wang, Z., Cho, A., Agudelo, M., Barnes, C.O., Gazumyan, A., Finkin, S., et al. (2020). Convergent antibody responses to SARS-CoV-2 in convalescent individuals. Nature *584*, 437–442.

Rogers, T.F., Zhao, F., Huang, D., Beutler, N., Burns, A., He, W.T., Limbo, O., Smith, C., Song, G., Woehl, J., et al. (2020). Isolation of potent SARS-CoV-2 neutralizing antibodies and protection from disease in a small animal model. Science *369*, 956–963.

Rosa, A., Pye, V.E., Graham, C., Muir, L., Seow, J., Ng, K.W., Cook, N.J., Rees-Spear, C., Parker, E., Silva Dos Santos, M., et al. (2021). SARS-CoV-2 can recruit a haem metabolite to evade antibody immunity. Sci. Adv. *7*, eabg7607.

Sadoff, J., Gray, G., Vandebosch, A., Cardenas, V., Shukarev, G., Grinsztejn, B., Goepfert, P.A., Truyers, C., Fennema, H., Spiessens, B., et al. (2021). Safety and efficacy of single-dose Ad26.COV2.S vaccine against covid-19. N. Engl. J. Med. *384*, 2187–2201.

Scheid, J.F., Mouquet, H., Feldhahn, N., Seaman, M.S., Velinzon, K., Pietzsch, J., Ott, R.G., Anthony, R.M., Zebroski, H., Hurley, A., et al. (2009). Broad diversity of neutralizing antibodies isolated from memory B cells in HIV-infected individuals. Nature *458*, 636–640.

Seow, J., Graham, C., Merrick, B., Acors, S., Pickering, S., Steel, K.J.A., Hemmings, O., O'Byrne, A., Kouphou, N., Galao, R.P., et al. (2020). Longitudinal observation and decline of neutralizing antibody responses in the three months following SARS-CoV-2 infection in humans. Nat. Microbiol. *5*, 1598–1607.

Seydoux, E., Homad, L.J., MacCamy, A.J., Parks, K.R., Hurlburt, N.K., Jennewein, M.F., Akins, N.R., Stuart, A.B., Wan, Y.H., Feng, J., et al. (2020). Analysis of a SARS-CoV-2-infected individual reveals development of potent neutralizing antibodies with limited somatic mutation. Immunity *53*, 98–105.e105.

Siu, J.H.Y., Pitcher, M.J., Tull, T.J., Velounias, R.L., Guesdon, W., Montorsi, L., Mahbubani, K.T., Ellis, R., Dhami, P., Todd, K., et al. (2022). Two subsets of human marginal zone B cells resolved by global analysis of lymphoid tissues and blood. Sci. Immunol. *7*, eabm9060.

Supasa, P., Zhou, D., Dejnirattisai, W., Liu, C., Mentzer, A.J., Ginn, H.M., Zhao, Y., Duyvesteyn, H.M.E., Nutalai, R., Tuekprakhon, A., et al. (2021). Reduced

neutralization of SARS-CoV-2 B.1.1.7 variant by convalescent and vaccine sera. Cell *184*, 2201–2211.e2207.

Suryadevara, N., Shrihari, S., Gilchuk, P., VanBlargan, L.A., Binshtein, E., Zost, S.J., Nargi, R.S., Sutton, R.E., Winkler, E.S., Chen, E.C., et al. (2021). Neutralizing and protective human monoclonal antibodies recognizing the N-terminal domain of the SARS-CoV-2 spike protein. Cell *184*, 2316–2331.e2315.

ter Meulen, J., van den Brink, E.N., Poon, L.L., Marissen, W.E., Leung, C.S., Cox, F., Cheung, C.Y., Bakker, A.Q., Bogaards, J.A., van Deventer, E., et al. (2006). Human monoclonal antibody combination against SARS coronavirus: synergy and coverage of escape mutants. PLoS Med. *3*, e237.

Thompson, C.P., Grayson, N.E., Paton, R.S., Bolton, J.S., Lourenco, J., Penman, B.S., Lee, L.N., Odon, V., Mongkolsapaya, J., Chinnakannan, S., et al. (2020). Detection of neutralising antibodies to SARS-CoV-2 to determine population exposure in Scottish blood donors between March and May 2020. Euro Surveill. *25*, 2000685.

Tiller, T., Meffre, E., Yurasov, S., Tsuiji, M., Nussenzweig, M.C., and Wardemann, H. (2008). Efficient generation of monoclonal antibodies from single human B cells by single cell RT-PCR and expression vector cloning. J. Immunol. Methods *329*, 112–124.

Tortorici, M.A., Beltramello, M., Lempp, F.A., Pinto, D., Dang, H.V., Rosen, L.E., McCallum, M., Bowen, J., Minola, A., Jaconi, S., et al. (2020). Ultrapotent human antibodies protect against SARS-CoV-2 challenge via multiple mechanisms. Science *370*, 950–957.

Turner, J.S., O'Halloran, J.A., Kalaidina, E., Kim, W., Schmitz, A.J., Zhou, J.Q., Lei, T., Thapa, M., Chen, R.E., Case, J.B., et al. (2021). SARS-CoV-2 mRNA vaccines induce persistent human germinal centre responses. Nature *596*, 109–113.

van den Brink, E.N., Ter Meulen, J., Cox, F., Jongeneelen, M.A., Thijsse, A., Throsby, M., Marissen, W.E., Rood, P.M., Bakker, A.B., Gelderblom, H.R., et al. (2005). Molecular and biological characterization of human monoclonal antibodies binding to the spike and nucleocapsid proteins of severe acute respiratory syndrome coronavirus. J. Virol. *79*, 1635–1644.

VanBlargan, L.A., Errico, J.M., Halfmann, P.J., Zost, S.J., Crowe, J.E., Jr., Purcell, L.A., Kawaoka, Y., Corti, D., Fremont, D.H., and Diamond, M.S. (2022). An infectious SARS-CoV-2 B.1.1.529 Omicron virus escapes neutralization by therapeutic monoclonal antibodies. Nat. Med. *28*, 490–495.

von Boehmer, L., Liu, C., Ackerman, S., Gitlin, A.D., Wang, Q., Gazumyan, A., and Nussenzweig, M.C. (2016). Sequencing and cloning of antigen-specific antibodies from mouse memory B cells. Nat. Protoc. *11*, 1908–1923.

Voysey, M., Clemens, S.A.C., Madhi, S.A., Weckx, L.Y., Folegatti, P.M., Aley, P.K., Angus, B., Baillie, V.L., Barnabas, S.L., Bhorat, Q.E., et al. (2021). Safety and efficacy of the ChAdOx1 nCoV-19 vaccine (AZD1222) against SARS-CoV-2: an interim analysis of four randomised controlled trials in Brazil, South Africa, and the UK. Lancet *397*, 99–111.

Wall, E.C., Wu, M., Harvey, R., Kelly, G., Warchal, S., Sawyer, C., Daniels, R., Adams, L., Hobson, P., Hatipoglu, E., et al. (2021). AZD1222-induced neutralising antibody activity against SARS-CoV-2 Delta VOC. Lancet *398*, 207–209.

Walsh, E.E., Frenck, R.W., Jr., Falsey, A.R., Kitchin, N., Absalon, J., Gurtman, A., Lockhart, S., Neuzil, K., Mulligan, M.J., Bailey, R., et al. (2020). Safety and immunogenicity of two RNA-based covid-19 vaccine candidates. N. Engl. J. Med. *383*, 2439–2450.

Wang, L., Zhou, T., Zhang, Y., Yang, E.S., Schramm, C.A., Shi, W., Pegu, A., Oloniniyi, O.K., Henry, A.R., Darko, S., et al. (2021a). Ultrapotent antibodies against diverse and highly transmissible SARS-CoV-2 variants. Science *373*, eabh1766.

Wang, P., Casner, R.G., Nair, M.S., Wang, M., Yu, J., Cerutti, G., Liu, L., Kwong, P.D., Huang, Y., Shapiro, L., et al. (2021b). Increased resistance of SARS-CoV-2 variant P.1 to antibody neutralization. Cell Host Microbe *29*, 747–751.e744.

Wang, P., Nair, M.S., Liu, L., Iketani, S., Luo, Y., Guo, Y., Wang, M., Yu, J., Zhang, B., Kwong, P.D., et al. (2021c). Antibody resistance of SARS-CoV-2 variants B.1.351 and B.1.1.7. Nature *593*, 130–135.

Wang, Z., Schmidt, F., Weisblum, Y., Muecksch, F., Barnes, C.O., Finkin, S., Schaefer-Babajew, D., Cipolla, M., Gaebler, C., Lieberman, J.A., et al. (2021d). mRNA vaccine-elicited antibodies to SARS-CoV-2 and circulating variants. Nature *592*, 616–622.

Wibmer, C.K., Ayres, F., Hermanus, T., Madzivhandila, M., Kgagudi, P., Oosthuysen, B., Lambson, B.E., de Oliveira, T., Vermeulen, M., van der Berg, K., et al. (2021). SARS-CoV-2 501Y.V2 escapes neutralization by South African COVID-19 donor plasma. Nat. Med. *27*, 622–625.

Wickham, H. (2016). ggplot2: Elegant Graphics for Data Analysis (New York: Springer-Verlag).

Winstone, H., Lista, M.J., Reid, A.C., Bouton, C., Pickering, S., Galao, R.P., Kerridge, C., Doores, K.J., Swanson, C., and Neil, S. (2021). The polybasic cleavage site in the SARS-CoV-2 spike modulates viral sensitivity to Type I interferon and IFITM2. J. Virol. *95*. e02422–20.

Wu, M., Wall, E.C., Carr, E.J., Harvey, R., Townsley, H., Mears, H.V., Adams, L., Kjaer, S., Kelly, G., Warchal, S., et al. (2022). Three-dose vaccination elicits neutralising antibodies against omicron. Lancet *399*, 715–717.

Wu, N.C., Yuan, M., Liu, H., Lee, C.D., Zhu, X., Bangaru, S., Torres, J.L., Caniels, T.G., Brouwer, P.J.M., van Gils, M.J., et al. (2020). An alternative binding mode of IGHV3-53 antibodies to the SARS-CoV-2 receptor binding domain. Cell Rep. *33*, 108274.

Yuan, M., Liu, H., Wu, N.C., Lee, C.D., Zhu, X., Zhao, F., Huang, D., Yu, W., Hua, Y., Tien, H., et al. (2020a). Structural basis of a shared antibody response to SARS-CoV-2. Science *369*, 1119–1123.

Yuan, M., Liu, H., Wu, N.C., and Wilson, I.A. (2020b). Recognition of the SARS-CoV-2 receptor binding domain by neutralizing antibodies. Biochem. Biophys. Res. Commun. *538*, 192–203.

Yuan, M., Wu, N.C., Zhu, X., Lee, C.D., So, R.T.Y., Lv, H., Mok, C.K.P., and Wilson, I.A. (2020c). A highly conserved cryptic epitope in the receptor binding domains of SARS-CoV-2 and SARS-CoV. Science *368*, 630–633.

Zhou, D., Dejnirattisai, W., Supasa, P., Liu, C., Mentzer, A.J., Ginn, H.M., Zhao, Y., Duyvesteyn, H.M.E., Tuekprakhon, A., Nutalai, R., et al. (2021). Evidence of escape of SARS-CoV-2 variant B.1.351 from natural and vaccine-induced sera. Cell *184*, 2348–2361.e6.

Zhou, T., Tsybovsky, Y., Gorman, J., Rapp, M., Cerutti, G., Chuang, G.Y., Katsamba, P.S., Sampson, J.M., Schon, A., Bimela, J., et al. (2020). Cryo-EM structures of SARS-CoV-2 spike without and with ACE2 reveal a pH-dependent switch to mediate endosomal positioning of receptor-binding domains. Cell Host Microbe *28*, 867–879.e865.

Zufferey, R., Nagy, D., Mandel, R.J., Naldini, L., and Trono, D. (1997). Multiply attenuated lentiviral vector achieves efficient gene delivery in vivo. Nat. Biotechnol. *15*, 871–875.

STAR★METHODS

KEY RESOURCES TABLE

REAGENT or RESOURCE	SOURCE	IDENTIFIER
Antibodies		
Goat-anti-human-Fc-AP	Jackson	RRID: AB_2337608<br>Cat#:109-055-098
horse-anti-mouse-IgG-HRP	Cell Signaling Technology	Cat#: S7076
Mouse-anti-human IgG Fc-PE	Biolegend	RRID: AB_10895907<br>Cat#: 409304
anti-CD3-APC/Cy7	Biolegend	RRID: AB_10644011<br>Cat#: 344817
anti-CD8-APC-Cy7	Biolegend	RRID: AB_2044005<br>Cat#: 344713
anti-CD14-BV510	Biolegend	RRID: AB_2561379<br>Cat#: 301841
anti-CD19-PerCP-Cy5.5	Biolegend	RRID: AB_2275547<br>Cat#: 302229
anti-IgM-PE	Biolegend	RRID: AB_493006<br>Cat#: 314507
anti-IgD-Pacific Blue	Biolegend	RRID: AB_2561596<br>Cat#: 348223
anti-IgG-PeCy7	BD Biosciences	RRID: AB_10611712<br>Cat#: 561298
Streptavidin-Alexa-488	Thermofisher Scientific	RRID: AB_2315383<br>Cat#: S32354
Streptavidin-APC	Thermofisher Scientific	Cat#: S32362
Streptavidin-PE	Thermofisher Scientific	Cat#: S21388
Murinized mAb CR3009	This manuscript (van den Brink et al., 2005)	N/A
mAb CR3022	This manuscript (ter Meulen et al., 2006)	N/A
SARS-CoV-2 specific nAbs and non-nAbs	This manuscript and (Graham et al., 2021)	N/A
Bacterial and virus strains		
NEB® Stable Competent *E. coli*	New England Biolabs	Cat#: C3040H
SARS-CoV-2 Strain England 2 (England 02/<br>2020/407073)	Public Health England (PHE)	N/A
Biological samples		
PBMC and plasma from AZD1222<br>vaccinated individual	This manuscript	N/A
Chemicals, peptides, and recombinant proteins		
Polyethylenimine, Linear, MW 25000 (PEI<br>Max)	Polysciences, Inc	Cat#: 23966
Polyethylenimine Hydrochloride, Linear,<br>MW 4,000	Polysciences, Inc	Cat#: 24885
Recombinant S1 (WT, B.1.1.7, B.1.351,<br>B.1.617.2)	Peter Cherepanov (Crick) (Rosa et al., 2021)<br>and this manuscript	N/A
Recombinant NTD	Peter Cherepanov (Crick) (Rosa et al., 2021)	N/A
Recombinant SARS-CoV-2 RBD ((WT,<br>B.1.1.7, B.1.351, B.1.617.2)	(Seow et al., 2020) and this manuscript	N/A
Recombinant Stabilized SARS-CoV-2<br>Spike	Marit van Gils (Amsterdam)<br>(Brouwer et al., 2020)	N/A

(*Continued on next page*)

Continued

REAGENT or RESOURCE	SOURCE	IDENTIFIER
Recombinant SARS-CoV-2 Spike (biotinylated)	This manuscript	N/A
IdeS	Max Crispin (University of Southampton) (Dixon, 2014)	N/A
Recombinant S2 protein	SinoBiological	Cat#: 40590-V08B
Protein G agarose	GE Healthcare	Cat#: Cytiva 17-0618-02
HiTrap IMAC columns	GE Healthcare	Cat#: Cytiva 17-0921-04
HILOAD 16/600 SUPERDEX 200 PG	GE Healthcare	Cat#: 28989335
Strep-TactinXT Superflow 50% Suspension	IBA	Cat#: 2-4010-002
BioLock blocking solution	IBA	Cat#: 2-0205-050
Ni Sepharose® 6 Fast Flow	Cytiva	Cat#: GE17-5318-06
Bright-Glo Luciferase Assay System	Promega	Cat#: E2610
**Critical commercial assays**		
Q5® Site-Directed Mutagenesis Kit	New England Biolabs	Cat#: E0554
Bright-Glo luciferase kit	Promega	Cat#: E2610
Qiagen Multiplex PCR kit	Qiagen	Cat#: 206145
Phusion High-Fidelity DNA Polymerase	NEB	Cat#: E2611L
SuperScript III RT	Thermofisher Scientific	Cat#: 18080085
LIVE/DEAD Fixable Aqua Dead Cell Stain Kit	Thermofisher Scientific	Cat#: L34957
1-Step™ Ultra TMB-ELISA Substrate Solution	Thermofisher Scientific	Cat#: 34028
Phosphatase substrate	Sigma Aldrich	Cat#: S0942-200TAB
**Deposited data**		
mAb sequence data	This manuscript	Accession numbers Genbank: ON088359–ON088446
**Experimental models: Cell lines**		
FreeStyle™ 293F Cells	Thermofisher Scientific	Cat#: R79007
HEK293T/17	ATCC	ATCC® CRL-11268™
HeLa-ACE2	James Voss (Scripps), (Rogers et al., 2020)	N/A
Vero-E6 TMPRSS2 cells	Stuart Neil	N/A
HEK293T	ATCC	ATCC® CRL-3216™
**Oligonucleotides**		
Heavy, kappa and Lambda PCR1 and 2 primers	(Scheid et al., 2009; Tiller et al., 2008; von Boehmer et al., 2016)	N/A
Spike mutagenesis primers	This manuscript	N/A
**Recombinant DNA**		
Biotinylated Spike (pHLSec)	This manuscript	N/A
Pre-fusion, stabilized and uncleaved SARS-CoV-2 Spike (pcDNA3.1+)	Marit van Gils (Amsterdam) (Brouwer et al., 2020)	N/A
Full length SARS-CoV-2 Spike (pcDNA3.1+)	Nigel Temperton (Seow et al., 2020)	N/A
Full length B.1.1.7 variant Spike (pcDNA3.1+)	Laura Mccoy (UCL) (Rees-Spear et al., 2021)	N/A
Full length P.1 variant Spike (pcDNA3.1+)	(Dupont et al., 2021)	N/A
Full length B.1.351 variant Spike (pcDNA3.1+)	(Dupont et al., 2021)	N/A
Full length B.1.617.2 variant Spike (pcDNA3.1+)	Wendy Barclay and (Dupont et al., 2021)	N/A

(*Continued on next page*)

Continued

REAGENT or RESOURCE	SOURCE	IDENTIFIER
Full length B.1.1.529 variant Spike (pcDNA3.1+)	Wendy Barclay	N/A
BirA	Addgene (Howarth et al., 2008)	Cat#: 20856
pHIV-Luc (constructed by replacing GFP in pHR'SIN-SEW (PMID: 11975847) with HA-luciferase)	Luis Apolonia (KCL)	N/A
HIV 8.91 gag/pol packaging construct	p8.91 (Zufferey et al., 1997)	N/A
Heavy/Kappa/Lambda human IgG1 expression vectors	M. Nussenzweig (Rockefeller University) von Boehmer et al., 2016)	N/A
Software and algorithms		
FlowJo	Tree Star	https://www.flowjo.com
Prism	Graphpad	https://www.graphpad.com/scientific-software/prism/
Tableau	TABLEAU SOFTWARE, LLC	https://www.tableau.com/
IMGT/V-QUEST	IMGT (Lefranc et al., 1999)	http://www.imgt.org/IMGT_vquest/vquest
R statistical programming environment	R Foundation for Statistical Computing	https://www.r-project.org
R studio	RStudio	https://www.rstudio.com/
ggplot2	(Wickham, 2016)	https://ggplot2.tidyverse.org
PyMol	The PyMOL Molecular Graphics System, Version 2.0 Schrödinger, LLC	https://www.pymol.org/
Other		
FACS Melody	BD Biosciences	N/A
Victor^{TM} X3 multilabel reader	Perkin Elmer	N/A

RESOURCE AVAILABILITY

Lead contact

Further information and requests for resources and reagents should be directed to and will be fulfilled by the lead contact, Katie J Doores (katie.doores@kcl.ac.uk).

Materials availability

Reagents generated in this study are available from the lead contact with a completed Materials Transfer Agreement.

Data and code availability

The antibody sequences generated during this study are available at GenBank (accession numbers Genbank: ON088359–ON088446).

This paper does not report original code.

Any additional information required to reanalyze the data reported in this paper is available from the lead contact upon request.

EXPERIMENTAL MODEL AND SUBJECT DETAILS

Ethics

This study used human samples from one donor collected as part of a study entitled "Antibody responses following COVID-19 vaccination". Ethical approval was obtained from the King's College London Infectious Diseases Biobank (IBD) (KDJF-110121) under the terms of the IDB's ethics permission (REC reference: 19/SC/0232) granted by the South Central – Hampshire B Research Ethics Committee in 2019. VA14 is male and 23 aged years.

Bacterial strains and cell culture

SARS-CoV-2 pseudotypes were produced by transfection of HEK293T/17 cells and neutralization activity assayed using HeLa cells stably expressing ACE2 (kind gift James E Voss). Small and large scale expression of monoclonal antibodies was performed in HEK293T/17 (ATCC; ATCC® CRL-11268^{TM}) and 293 Freestyle cells (Thermofisher Scientific), respectively. Bacterial transformations were performed with NEB® Stable Competent *E. coli*.

METHOD DETAILS

Protein expression and purification

Recombinant Spike and RBD for ELISA were expressed and purified as previously described (Pickering et al., 2020; Seow et al., 2020). Recombinant S1 (residues 1-530) and NTD (residues 1-310) expression and purification was described in Rosa et al. (Rosa et al., 2021). S2 protein was obtained from SinoBiological (Cat number: 40590-V08B).

For antigen-specific B cell sorting, Spike glycoprotein consisted of the pre-fusion S ectodomain (residues 1–1138) with a GGGG substitution at the furin cleavage site (amino acids 682–685), proline substitutions at amino acid positions 986 and 987, and an N-terminal T4 trimerization domain. RBD consisted of amino acids 331-533. Spike and RBD were cloned into a pHLsec vector containing Avi and 6xHis tags (Aricescu et al., 2006). Biotinylated Spike or RBD were expressed in 1L of HEK293F cells (Invitrogen) at a density of 1.5×10^6 cells/mL. To achieve *in vivo* biotinylation, 480μg of each plasmid was co-transfected with 120μg of BirA (Howarth et al., 2008) and 12mg PEI-Max (1 mg/mL solution, Polysciences) in the presence of 200 μM biotin (final concentration). The supernatant was harvested after 7 days and purified using immobilized metal affinity chromatography and size-exclusion chromatography. Complete biotinylation was confirmed via depletion of protein using avidin beads.

ELISA (S, RBD, NTD, S2 or S1)

96-well plates (Corning, 3690) were coated with S, S1, NTD, S2 or RBD at 3 μg/mL overnight at 4°C. The plates were washed (5 times with PBS/0.05% Tween-20, PBS-T), blocked with blocking buffer (5% skimmed milk in PBS-T) for 1 h at room temperature. Serial dilutions of plasma, mAb or supernatant in blocking buffer were added and incubated for 2 hr at room temperature. Plates were washed (5 times with PBS-T) and secondary antibody was added and incubated for 1 hr at room temperature. IgM was detected using Goat-anti-human-IgM-HRP (horseradish peroxidase) (1:1,000) (Sigma: A6907) and IgG was detected using Goat-anti-human-Fc-AP (alkaline phosphatase) (1:1,000) (Jackson: 109-055-098). Plates were washed (5 times with PBS-T) and developed with either AP substrate (Sigma) and read at 405 nm (AP) or 1-step TMB (3,3′,5,5′-Tetramethylbenzidine) substrate (Thermo Scientific) and quenched with 0.5 M H_2S0_4 before reading at 450 nm (HRP).

Biliverdin competition ELISA

ELISA plates were coated with 3 μg/ml (25 μl per well) SARS-CoV2 WT S1 antigen in PBS overnight at 4°C. Wells were blocked with 100 μl 2% casein in PBS for 1 h at room temperature. The wells were emptied and 25 μl of 2% casein in PBS was added per well. This solution was supplemented with biliverdin at 10 μM where indicated. Serial dilutions of IgGs were prepared in separate 96-well plate in 2% casein, and then 25 μl of each serial dilution added to the ELISA assay plates and incubated for 2 h at room temperature. Wells were washed with PBS-T. IgG binding was detected using goat-anti-human-Fc conjugated to alkaline phosphatase (1:1,000; Jackson, product code 109-055-098). Wells were washed with PBS-T and alkaline phosphatase substrate (Sigma-Aldrich) was added and read at 405 nm.

Fab/Fc ELISA

96-well plates (Corning, 3690) were coated with goat anti-human Fc IgG antibody at 3 μg/mL overnight at 4°C. The above protocol was followed. The presence of IgG in supernatants was detected using Goat-anti-human-Fc-AP (alkaline phosphatase) (1:1,000) (Jackson: 109-055-098).

IgG digestion to generate F(ab’)$_2$

IgG were incubated with IdeS (Dixon, 2014) (4 μg of IdeS per 1 mg of IgG) in PBS for 1 hour at 37°C. The Fc and IdeS A were removed using a mix of Protein A Sepharose® Fast Flow (250 μL per 1 mg digested mAb; GE Healthcare Life Sciences) and Ni Sepharose™ 6 Fast Flow (50 μL per 1 mg digested mAb; GE Healthcare Life Sciences) which were washed twice with PBS before adding to the reaction mixture. After exactly 10 minutes the beads were removed from the F(ab’)$_2$-dilution by filtration in Spin-X tube filters (Costar®) and the filtrate was concentrated in Amicon® Ultra Filters (10k, Millipore). Purified F(ab’)$_2$ fragments were analysed by SDS-PAGE.

F(ab’)$_2$ and IgG competition ELISA

96-well half area high bind microplates (Corning®) were coated with S-protein at 3 μg/mL in PBS overnight at 4°C. Plates were washed (5 times with PBS/0.05% Tween-20, PBS-T) and blocked with 5% milk in PBS/T for 2 hr at room temperature. Serial dilutions (5-fold) of F(ab’)$_2$, starting at 100-molar excess of the IC_{80} of S binding, were added to the plates and incubated for 1 hr at room temperature. Plates were washed (5 times with PBS-T) before competing IgG was added at their IC_{80} of S binding and incubated at room temperature for 1 hr. Plates were washed (5 times with PBS-T) and Goat-anti-human-Fc-AP (alkaline phosphatase) (1:1,000) (Jackson: 109-055-098) was added and incubated for 45 minutes at room temperature. The plates were washed (5 times with PBS-T) and AP substrate (Sigma) was added. Optical density was measured at 405 nm in 5-minute intervals. The percentage competition was calculated as the reduction in IgG binding in the presence of F(ab’)$_2$ (at 100-molar excess of the IC_{80}) as a percentage of the maximum IgG binding in the absence of F(ab’)$_2$. Competition groups were determined using Ward2 clustering (R, Complex Heatmap package (Gu et al., 2016)) for initial analysis and Groups were then arranged by hand according to binding epitopes.

Semi-quantitative ELISA

In 96-well plates (Corning, 3690), 10 columns were coated with SARS-CoV-2 Spike at 3 μg/mL in PBS, with the remaining 2 columns coated with Goat anti-Human IgG F(ab')$_2$ at 1:1000 dilution, and incubated overnight at 4°C. The plates were washed (5 times with PBS/0.05% Tween-20, PBS-T) and blocked with blocking buffer (5% skimmed milk in PBS-T) for 1 h at room temperature. Serial dilutions of serum and a known concentrations of IgG standard (in blocking buffer) were added to the Spike coated and standard curve columns, respectively. After 2 h incubation at room temperature, plates were washed 5 times with PBS-T. Secondary antibody, goat-anti-human-Fc-AP, was added at 1:1000 dilution in blocking buffer and incubated for 1 h at room temperature. Plates were washed 5 times with PBS-T and developed with AP substrate (Sigma). Absorbance was measured at 405 nm. Antigen-specific serum IgG was quantified by averaging values interpolated from a standard curve of IgG standard using four-parameter logistic regression curve fitting (Rees-Spear et al., 2021).

SARS-CoV-2 pseudotyped virus preparation

Pseudotyped HIV-1 virus incorporating either the SARS-Cov-2 Wuhan, B.1.1.7, P.1, B.1.351, B.1.617.2, B.1.1.529 full-length Spike were produced in a 10 cm dish seeded the day prior with 5x10^6 HEK293T/17 cells in 10 mL of complete Dulbecco's Modified Eagle's Medium (DMEM-C, 10% fetal bovine serum (FBS) and 1% Pen/Strep (100 IU/mL penicillin and 100 mg/mL streptomycin)). Cells were transfected using 90 mg of PEI-Max (1 mg/mL, Polysciences) with: 15 μg of HIV-luciferase plasmid, 10 μg of HIV 8.91 gag/pol plasmid (Zufferey et al., 1997) and 5 μg of SARS-CoV-2 spike protein plasmid (Grehan et al., 2015; Thompson et al., 2020). Pseudotyped virus was harvested after 72 hours, filtered through a 0.45mm filter and stored at -80°C until required.

Neutralization assay with SARS-CoV-2 pseudotyped virus

Neutralization assays were conducted as previously described (Carter et al., 2020; Monin et al., 2021; Seow et al., 2020). Serial dilutions of serum samples (heat inactivated at 56°C for 30mins) or mAbs were prepared with DMEM-C media and incubated with pseudotyped virus for 1-hour at 37°C in 96-well plates. Next, HeLa cells stably expressing the ACE2 receptor (provided by Dr James Voss, Scripps Research, La Jolla, CA) were added (12,500 cells/50μL per well) and the plates were left for 72 hours. The amount of infection was assessed in lysed cells with the Bright-Glo luciferase kit (Promega), using a Victor™ X3 multilabel reader (Perkin Elmer). Measurements were performed in duplicate and duplicates used to calculate the ID_{50}.

Infectious virus strain and propagation

Vero-E6 TMPRSS2 cells (Winstone et al., 2021) (Cercopithecus aethiops derived epithelial kidney cells) cells were grown in Dulbecco's modified Eagle's medium (DMEM, Gibco) supplemented with GlutaMAX, 10% fetal bovine serum (FBS), 20 μg/mL gentamicin, and incubated at 37°C with 5% CO_2. SARS-CoV-2 Strain England 2 (England 02/2020/407073) was obtained from Public Health England. The virus was propagated by infecting 60-70% confluent Vero-E6 TMPRSS2 cells in T75 flasks, at an MOI of 0.005 in 3 mL of DMEM supplemented with GlutaMAX and 10% FBS. Cells were incubated for 1 hr at 37°C before adding 15 mL of the same medium. Supernatant was harvested 72h post-infection following visible cytopathic effect (CPE), and filtered through a 0.22 μm filter to eliminate debris, aliquoted and stored at -80C. The infectious virus titre was determined by plaque assay using Vero-E6 TMPRSS2 cells.

Infectious virus neutralization assay

Vero-E6 TMPRSS2 cells (Winstone et al., 2021) were seeded at a concentration of 20,000 cells/100uL per well in 96-well plates in DMEM media (10% FBS and 1% Pen/Strep) and allowed to adhere overnight. Serial dilutions of mAbs were prepared with DMEM media (2% FBS and 1% Pen/Strep) and incubated with replication competent live SARS-CoV-2 for 1 hour at 37°C. The media was removed from the pre-plated Vero-E6 TMPRSS2 cells and the serum-virus mixtures were added to the cells and incubated at 37°C for 24 h. The virus/serum mixture was aspirated, and each well was fixed with 150μL of 4% formalin at 4°C overnight and then topped up to 300μL using PBS. The cells were washed once with PBS and permeabilized with 0.1% Triton-X in PBS at room temperature for 15 min. The cells were washed twice with PBS and blocked using 3% milk in PBS at room temperature for 15 min. The blocking solution was removed and an N-specific mAb (murinized-CR3009 (van den Brink et al., 2005)) was added at 2 μg/mL (diluted using 1% milk in PBS) at room temperature for 45 min. The cells were washed twice with PBS and goat-anti-mouse-IgG-conjugated to HRP was added (1:2000 in 1% milk in PBS, A2554-1mL, Sigma-Aldrich) at room temperature for 1 hour. The cells were washed twice with PBS, developed using TMB substrate for 30 min and quenched using 2M H_2SO_4 prior to reading at 450 nm. Measurements were performed in duplicate and the duplicates used to calculate the ID_{50}.

Antigen-specific B cell sorting

Fluorescence-activated cell sorting of cryopreserved PBMCs was performed on a BD FACS Melody as previously described (Graham et al., 2021). Sorting baits (SARS-CoV-2 Spike and RBD) was pre-complexed with the streptavidin fluorophore at a 1:4 molar ratio prior to addition to cells. PBMCs were stained with live/dead (fixable Aqua Dead, Thermofisher), anti-CD3-APC/Cy7 (Biolegend), anti-CD8-APC-Cy7 (Biolegend), anti-CD14-BV510 (Biolegend), anti-CD19-PerCP-Cy5.5 (Biolegend), anti-IgM-PE (Biolegend), anti-IgD-Pacific Blue (Biolegend) and anti-IgG-PeCy7 (BD) and Spike-Alexa488 (Thermofisher Scientific, S32354) and Spike-APC (Thermofisher Scientific, S32362) or RBD-Alexa488 and RBD-APC. Live $CD3/CD8^-CD14^-CD19^+IgM^-IgD^-IgG^+Spike^+Spike^+$ or $CD3/CD8^-CD14^-CD19^+IgM^-IgD^-IgG^+RBD^+RBD^+$ cells were sorted using a BD FACS Melody into individual wells containing RNase

OUT (Invitrogen), First Strand SuperScript III buffer, DTT and H_2O (Invitrogen) and RNA was converted into cDNA (SuperScript III Reverse Transcriptase, Invitrogen) using random hexamers (Bioline Reagents Ltd) following the manufacturer's protocol.

Full-length antibody cloning and expression

The human Ab variable regions of heavy and kappa/lambda chains were PCR amplified using previously described primers and PCR conditions (Scheid et al., 2009; Tiller et al., 2008; von Boehmer et al., 2016). PCR products were purified and cloned into human-IgG (Heavy, Kappa or Lambda) expression plasmids(von Boehmer et al., 2016) using the Gibson Assembly Master Mix (NEB) following the manufacturer's protocol. Gibson assembly products were directly transfected into HEK-293T cells and transformed under ampicillin selection. Ab supernatants were harvested 3 days after transfection and IgG expression and Spike-reactivity determined using ELISA. Ab variable regions of heavy-light chain pairs that generated Spike reactive IgG were sequenced by Sanger sequencing.

IgG expression and purification

Ab heavy and light plasmids were co-transfected at a 1:1 ratio into HEK-293F cells (Thermofisher) using PEI Max (1 mg/mL, Polysciences, Inc.) at a 3:1 ratio (PEI Max:DNA). Ab supernatants were harvested five days following transfection, filtered and purified using protein G affinity chromatography following the manufacturer's protocol (GE Healthcare).

ACE2 competition measured by flow cytometry

To prepare the fluorescent probe, Streptavidin-APC (Thermofisher Scientific, S32362) was added to biotinylated SARS-CoV-2 Spike (3.5 times molar excess of Spike) on ice. Additions were staggered over 5 steps with 30 min incubation times between each addition. Purified mAbs were mixed with PE conjugated SARS-CoV-2 S in a molar ratio of 4:1 in FACS buffer (2% FBS in PBS) on ice for 1 h. HeLa-ACE2 and HeLa cells were washed once with PBS and detached using PBS containing 5mM EDTA. Detached cells were washed and resuspended in FACS buffer. 0.5 million HeLa-ACE2 cells were added to each mAb-Spike complex and incubated on ice for 30 m. The cells were washed with PBS and resuspended in 1 mL FACS buffer with 1 μL of LIVE/DEAD Fixable Aqua Dead Cell Stain Kit (Invitrogen). HeLa-ACE2 cells alone and with SARS-CoV-2 Spike only were used as background and positive controls, respectively. The geometric mean fluorescence for PE was measured from the gate of singlet and live cells. The ACE2 binding inhibition percentage was calculated as described previously (Graham et al., 2021; Rogers et al., 2020).

Monoclonal antibody sequence analysis

The heavy and light chain sequences of SARS-CoV-2 specific mAbs were examined using IMGT/V-QUEST (http://www.imgt.org/IMGT_vquest/vquest) to identify the germline usages, percentage of SHM and CDR region lengths. To remove variation introduced through cloning using mixture of forward primers, 5 amino acids or 15 nucleotides were trimmed from the start and end of the translated variable genes. D'Agostino & Pearson normality test, Kruskal-Wallis test with Dunn's multiple comparisons post hoc test, Ordinary one-way ANOVA with Tukey's multiple comparisons post hoc test and two-sided binomial tests) were performed using GraphPad Prism software. Significance defined as $p < 0.0332$ (*), 0.0021 (**), 0.0002 (***) and >0.0001 (****).

QUANTIFICATION AND STATISTICAL ANALYSIS

All neutralization and ELISA experiments were performed in duplicate. The 50% inhibitory concentrations/dilutions (IC/ID_{50}) were calculated using GraphPad Prism software. Statistical analysis in Figures 3 and S2 (D'Agostino & Pearson tests was performed to determine normality and Kruskal-Wallis with Dunn's multiple comparison test post hoc test or an ordinary one-way ANOVA with Turkey's multiple comparison post hoc) were performed using GraphPad Prism software, significance defined as $p<0.05$. Linear correlations (Figure S2, Spearman correlation) were also calculated using GraphPad Prism. The fold decrease in mAb IC_{50} was calculated by dividing the average IC_{50} value for a given mAb against the indicated VOC by the IC_{50} value for that mAb against the WT (Figure 6).

Cell Reports, Volume *39*

Supplemental information

ChAdOx1 nCoV-19 vaccine elicits monoclonal antibodies with cross-neutralizing activity against SARS-CoV-2 viral variants

Jeffrey Seow, Carl Graham, Sadie R. Hallett, Thomas Lechmere, Thomas J.A. Maguire, Isabella Huettner, Daniel Cox, Hataf Khan, Suzanne Pickering, Rebekah Roberts, Anele Waters, Christopher C. Ward, Christine Mant, Michael J. Pitcher, Jo Spencer, Julie Fox, Michael H. Malim, and Katie J. Doores

ChAdOx1 nCoV-19 (AZD1222) vaccine elicits monoclonal antibodies with potent cross-neutralizing activity against SARS-CoV-2 viral variants

Jeffrey Seow,[1]* Carl Graham,[1]* Sadie R. Hallett,[1] Thomas Lechmere,[1] Thomas J.A. Maguire,[1] Isabella Huettner,[1] Daniel Cox,[1] Hataf Khan,[1] Suzanne Pickering,[1] Rebekah Roberts,[2] Anele Waters,[2] Christopher C. Ward,[1] Christine Mant,[3] Michael J. Pitcher,[4] Jo Spencer,[4] Julie Fox,[1,2] Michael H. Malim,[1] Katie J. Doores[1]#

[1] Department of Infectious Diseases, School of Immunology & Microbial Sciences, King's College London, London, UK.
[2] Harrison Wing, Guys and St Thomas' NHS Trust, London, UK.
[3] Infectious Diseases Biobank, Department of Infectious Diseases, School of Immunology and Microbial Sciences, King's College London, London, UK.
[4] Peter Gorer Department of Immunobiology, School of Immunology & Microbial Sciences, King's College London, London, UK.

* These authors contributed equally
\# Corresponding author: katie.doores@kcl.ac.uk

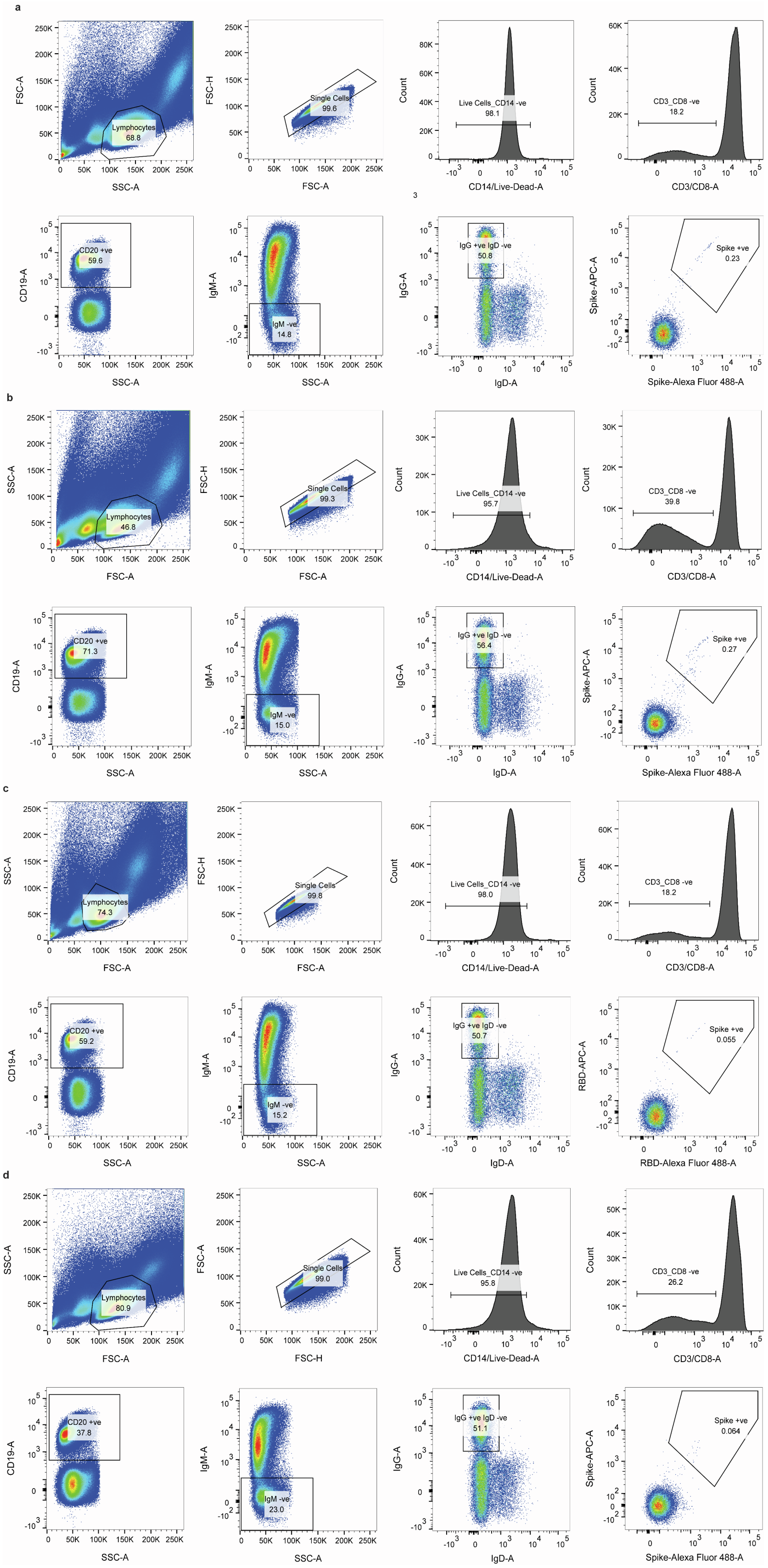

Supplementary Figure 1: FACS sorting strategy to isolate Spike and RBD reactive mAbs following vaccination with AZD1222, related to **Figure 1**. Sorting of Spike reactive IgG+ B cells from VA14 at **A)** 4-months (TP1) and **B)** 9-months (TP2) post vaccine booster. **C)** Sorting of RBD reactive IgG+ B cells from VA14 at 4-months post vaccine booster. **D)** Staining of PBMC collecting pre-COVID-19 from a healthy control.

a

Name	Protein + Epitope	VH-GENE	JH-GENE	DH-GENE	VL-GENE	JL-GENE	CDRH3 Length	CDRL3 Length	Neutralzation	% Identity Heavy	% Identity Light
VA014_09	RBD	IGHV1-18	IGHJ3	IGHD3-10	IGKV3-11	IGKJ4	16	8	Yes	95.41	96.55
VA014_50	RBD	IGHV1-18	IGHJ3	IGHD3-10	IGKV3-11	IGKJ4	16	8	Yes		
VA014_R39	RBD	IGHV4-34	IGHJ6	IGHD4-17	IGKV1-5	IGKJ2	18	8	Yes	87.1	94.36
VA014_26	RBD	IGHV4-34	IGHJ6	IGHD4-17	IGKV1-5	IGKJ2	18	8	Yes		
VA014_27	RBD	IGHV5-51	IGHJ3	IGHD2-2	IGKV1-39	IGKJ2	23	9	Yes	95.38	95.03
VA014_67	RBD	IGHV5-51	IGHJ6*	IGHD2-2	IGKV1-39	IGKJ2	23	9	Yes		

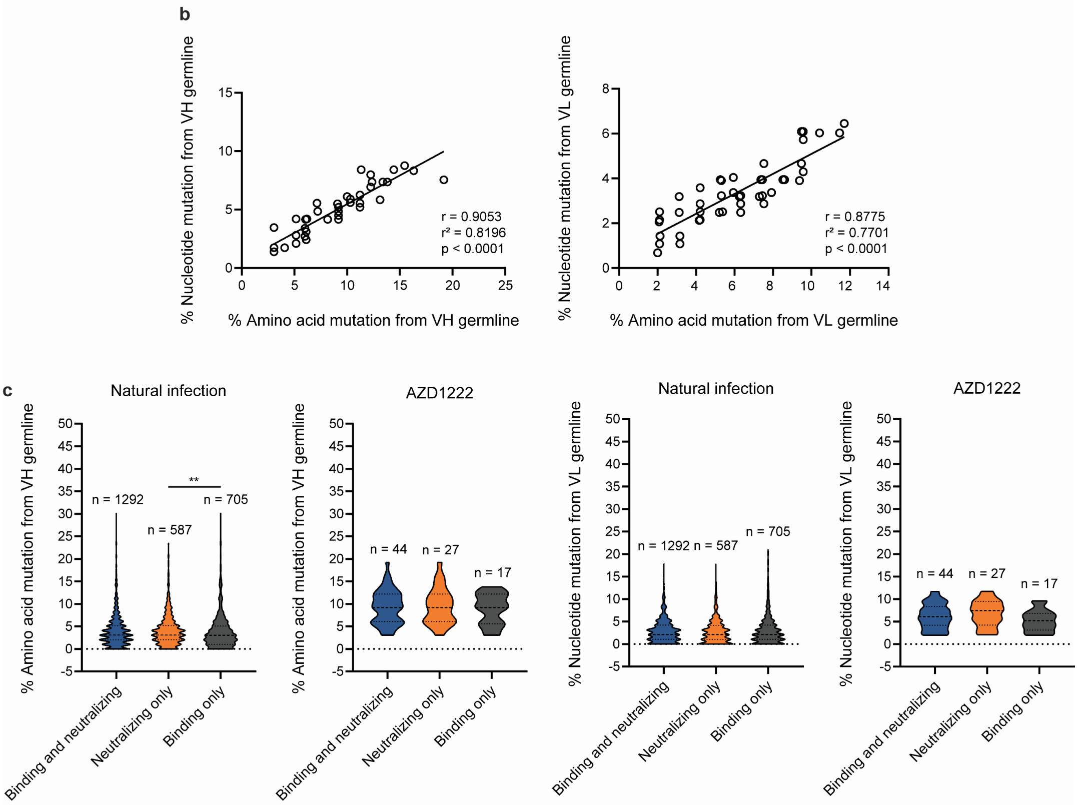

Supplementary Figure 2: Sequence analysis of AZD1222 elicited mAbs, related to **Figure 3**. **A)** Clonally related mAbs isolated from VA14. **B)** Correlation between the % nucleotide mutation and % amino acid mutation for VH and VL germline for AZD1222 elicited mAbs (Spearman correlation, two-tailed, r). A linear regression was used to calculate the goodness of fit (r^2). **C)** Divergence from germline (based on amino acid alignments) for VH and VL genes for Spike reactive mAbs arising from natural infection and AZD1222 vaccination. Spike reactive mAbs have been separated based on their binding and/or neutralizing properties. D'Agostino & Pearson tests were performed on each dataset to determine normality. Based on the result, either a Kruskal-Wallis test with Dunn's multiple comparison post hoc test or an ordinary one-way ANOVA with Turkey's multiple comparison post hoc test was performed. *p<0.0332, **p<0.0021, ***p<0.0002 and ****<0.0001.

A: RBD mAbs with RBD ELISA

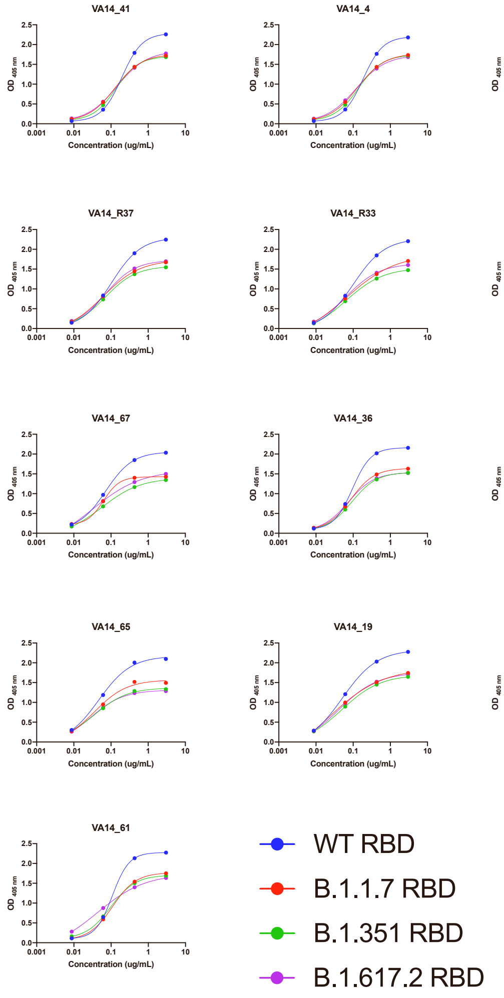

B: NTD mAbs with S1 ELISA

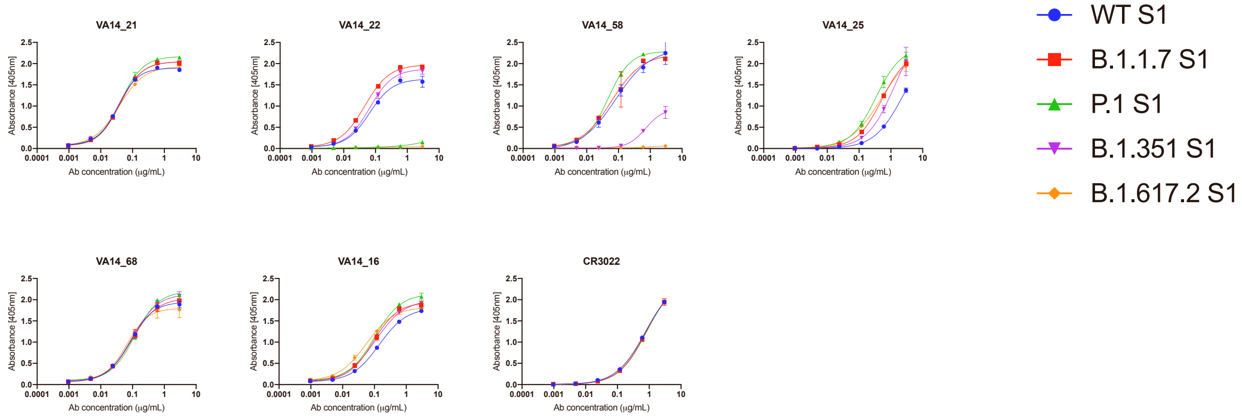

Supplementary Figure 3: Binding of AZD1222 neutralizing antibodies to RBD or S1 from SARS-CoV-2 variants of concern, related to **Figure 4** and **Figure 5**. **A)** Binding of RBD-specific nAbs to recombinant RBD from WT, alpha, beta and delta by ELISA. **B)** Binding of NTD-specific nAbs to recombinant S1 from WT, alpha, beta, gamma and delta by ELISA.

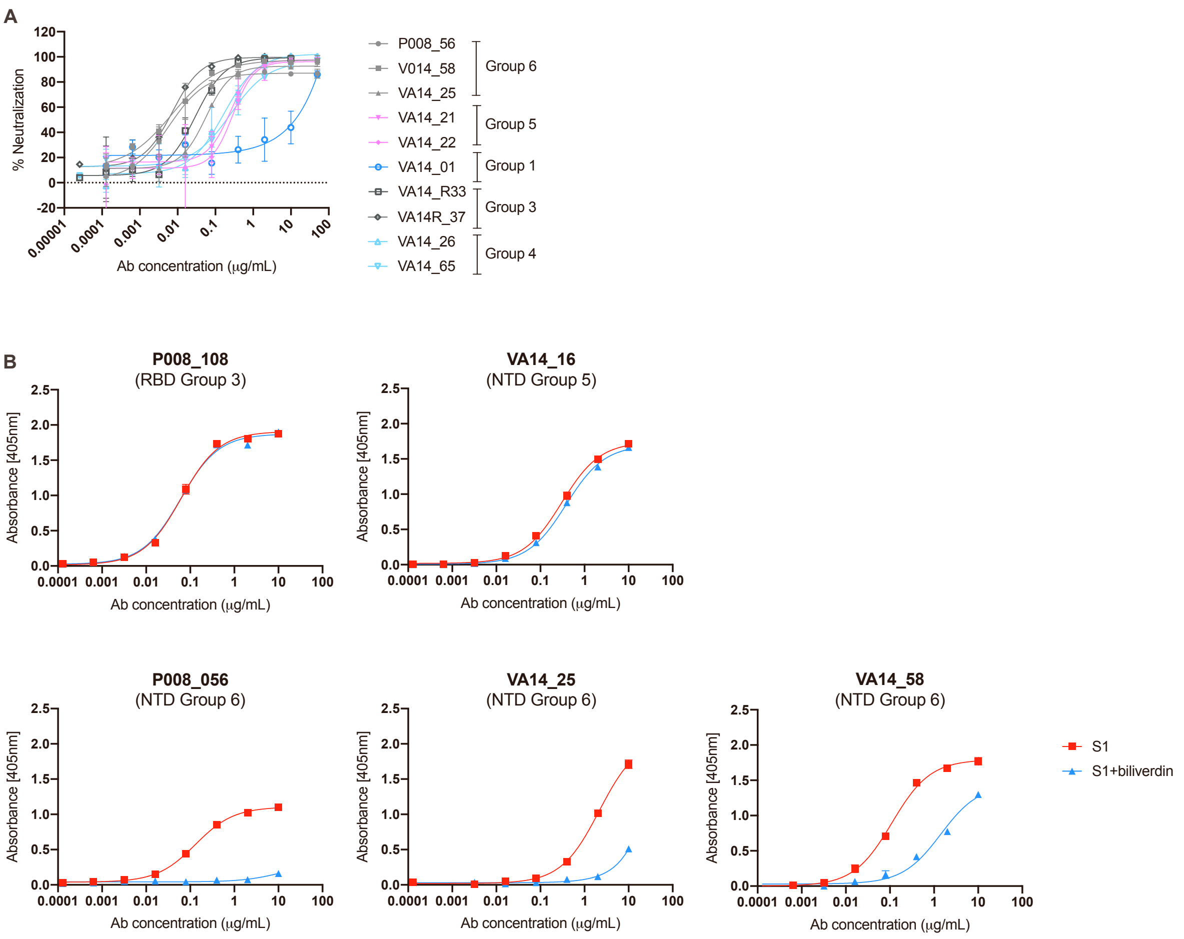

Supplemental Figure 4: Characterisation of NTD-specific Group 6 nAbs, related to **Figure 5**. **A)** Neutralization of SARS-CoV-2 (England 02/2020/407073) infectious virus using Vero-E6 TMPRSS2 cells. Experiments were performed in duplicate. nAbs are colour coded by group. **B)** Binding of VA14 NTD Group 6 mAbs to S1 in the presence (blue) and absence (red) of Biliverdin. RBD Group mAb P008_108 and NTD Group 5 mAb VA14_16 are used as negative controls. NTD Group 6 mAb P008_056 is used as a positive control (Rosa et al., 2021).

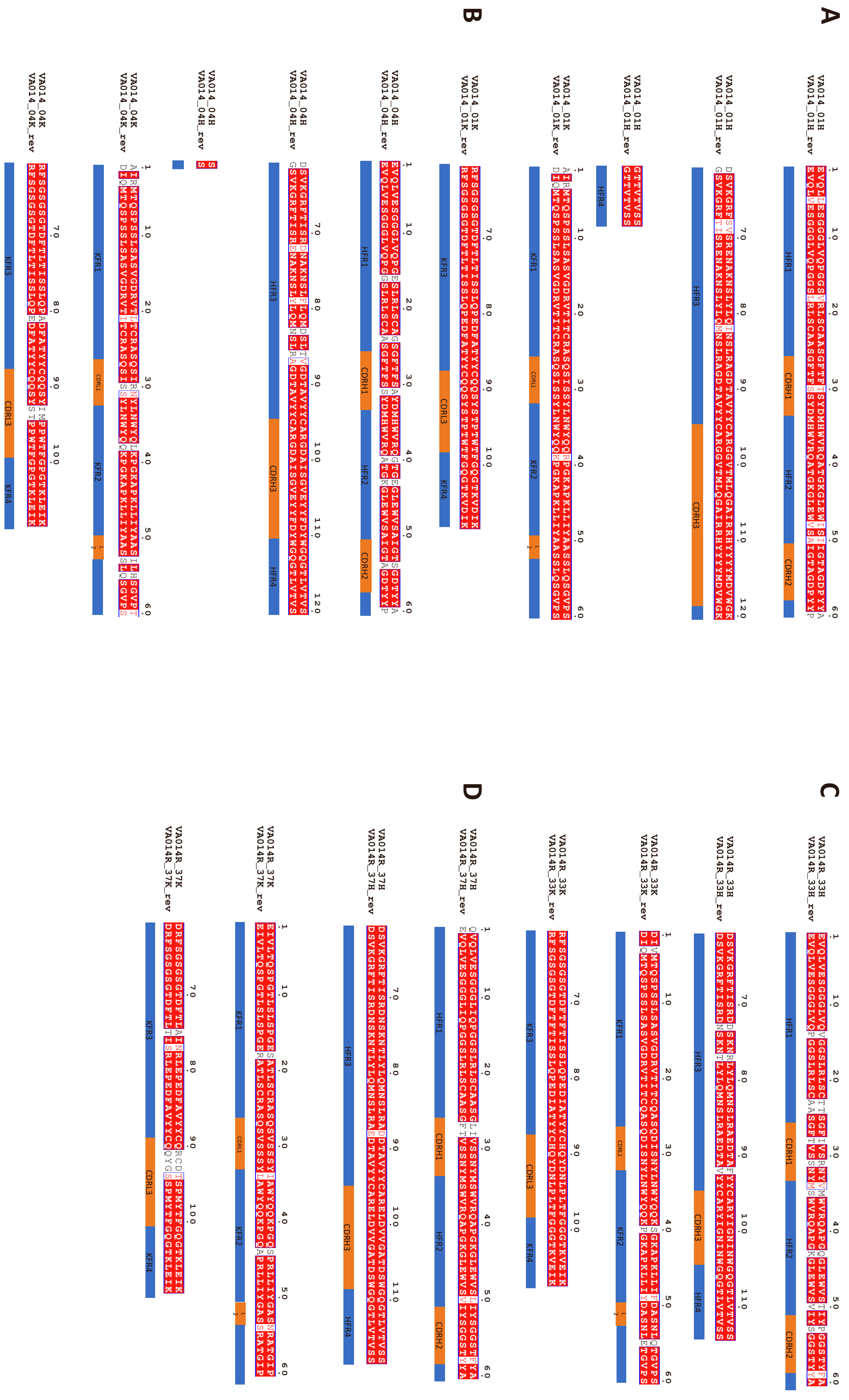

Supplementary Figure 5: Amino acid alignments for germline reverted heavy and light chains, related to **Figure 6**. **A)** VA14_01, **B)** VA14_04, **C)** VA14R_33, **D)** VA14R_37. Top germline V-gene hits were determined using the International Immunogenetics Information System (IMGT) database (Lefranc et al., 1999). The sequence alignment was determined by Clustal Omega (Sievers et al., 2011) and plotted with ESPRIPT (Robert and Gouet, 2014). CDR1, 2 and 3 regions are highlighted. Related to **Figure 6**.

	WT IC_{50}	B.1.1.7 IC_{50}	P.1 IC_{50}	B.1.351 IC_{50}	B.1.617.2 IC_{50}	B.1.1.529 IC_{50}	ACE2 Competition	Specificity	Competition Group	VH	VL
VA014_04	0.42	0.78	0.56	0.65	0.82	5.63	96	RBD	Group 1	IGHV3-13	IGKV1-39
VA014_41	0.84	1.46	0.69	1.26	1.52	48.12	91	RBD	Group 1	IGHV3-30	IGKV1-39
VA014_01	7.34	5.18	2.94	8.17	11.08	44.68	66	RBD	Group 1	IGHV3-13	IGKV1-39
VA014R_39	0.070	0.091	0.079	0.046	0.055	>50	99	RBD	Group 1	IGHV4-34	IGKV1-5
VA014_26	0.21	0.17	0.093	0.11	0.11	>50	98	RBD	Group 2	IGHV4-34	IGKV1-5
VA014R_37	0.024	0.0094	0.0038	0.0013	0.018	0.0020	99	RBD	Group 3	IGHV3-53	IGKV3-20
VA014R_33	0.010	0.020	0.0040	0.0011	0.018	0.0050	99	RBD	Group 3	IGHV3-66	IGKV1-33
VA014_43	0.029	0.0044	0.0037	0.0097	0.017	0.164	99	RBD	Group 3	IGHV3-53	IGKV1-9
VA014_33	0.056	0.031	0.0056	0.0056	0.013	0.010	90	RBD	Group 4	IGHV3-74	IGLV6-57
VA014_36	0.025	0.020	0.013	0.0062	0.020	1.26	72	RBD	Group 4	IGHV4-59	IGKV1-33
VA014_65	0.047	0.038	0.047	0.27	>50	0.19	59	RBD	Group 4	IGHV3-21	IGKV1-39
VA014_19	0.79	0.32	0.24	1.15	0.29	0.65	55	RBD	Group 4	IGHV3-15	IGKV1-39
VA014_49	3.06	0.91	1.31	3.04	2.16	5.98	55	RBD	Group 4	IGHV3-30	IGKV1-33
VA014R_38	0.038	0.040	0.053	0.092	0.040	0.12	45	RBD	Group 4	IGHV3-43	IGKV3-20
VA014_50	0.031	0.024	0.66	1.39	>50	1.01	45	RBD	Group 4	IGHV1-18	IGKV3-11
VA014_10	0.78	0.13	3.70	4.95	0.25	5.08	45	RBD	Group 4	IGHV3-23	IGKV1-33
VA014_67	0.029	0.022	0.049	0.29	0.055	0.52	43	RBD	Group 4	IGHV5-51	IGKV1-39
VA014_07	0.28	0.19	0.46	1.16	0.86	3.08	43	RBD	Group 4	IGHV4-39	IGKV3-20
VA014_27	0.24	0.18	0.16	0.73	1.98	0.96	34	RBD	Group 4	IGHV5-51	IGKV1-39
VA014_09	0.027	0.019	11.9	>50	>50	13.08	28	RBD	Group 4	IGHV1-18	IGKV3-11
VA014_61	0.071	0.016	0.0069	0.0060	0.032	0.0020	96	RBD	Group 4	IGHV1-69	IGKV4-1
VA014_21	1.22	0.84	13.4	5.17	14.07	3.17	58	NTD	Group 5	IGHV1-69	IGKV4-1
VA014_22	0.13	0.53	>50	0.78	>50	0.95	51	NTD	Group 5	IGHV3-48	IGKV1D-12
VA014_58	>50	0.0085	0.026	>50	>50	>50	28	NTD	Group 6	IGHV1-8	IGKV3-11
VA014_25	>50	0.052	0.093	>50	>50	>50	19	NTD	Group 6	IGHV1-69	IGKV3-15
VA014_47	0.86	32.37	>50	1.38	1.08	0.79	59	S only	Group 7	IGHV3-30	IGKV3-20
VA014_16	0.14	0.051	0.14	0.22	0.027	1.59	23	NTD	Group 8	IGHV3-48	IGKV1-17
VA014_68	0.39	0.075	10.19	0.48	0.067	1.86	19	NTD	Group 8	IGHV4-59	IGKV4-1

Supplementary Table S1: Binding and neutralization properties of isolated mAbs, related to **Figure 2** and **Figure 5**.
